# Systems Pharmacology Dissection of Multi-Scale Mechanisms of Action of *Huo-Xiang-Zheng-Qi* Formula for the Treatment of Gastrointestinal Diseases

**DOI:** 10.3389/fphar.2018.01448

**Published:** 2019-01-11

**Authors:** Miaoqing Zhao, Yangyang Chen, Chao Wang, Wei Xiao, Shusheng Chen, Shuwei Zhang, Ling Yang, Yan Li

**Affiliations:** ^1^Key Laboratory of Industrial Ecology and Environmental Engineering, Faculty of Chemical, Environmental and Biological Science and Technology, Dalian University of Technology, Dalian, China; ^2^Key Laboratory of Xinjiang Endemic Phytomedicine Resources, Pharmacy School, Shihezi University, Shihezi, China; ^3^Lab of Systems Pharmacology, Center of Bioinformatics, College of Life Sciences, Northwest A&F University, Yangling, China; ^4^State Key Laboratory of New-tech for Chinese Medicine Pharmaceutical Process, Jiangsu Kanion Pharmaceutical Co., Ltd., Lianyungang, China; ^5^Systems Biology Laboratory, Department of Computer & Information Science & Engineering, University of Florida, Gainesville, FL, United States; ^6^Institute of Interdisciplinary Integrative Medicine Research, Shanghai University of Traditional Chinese Medicine, Shanghai, China

**Keywords:** TCM, gastrointestinal diseases, functional dyspepsia, systems pharmacology, *Huo-xiang-zheng-qi*, compatibility theory

## Abstract

Multi-components Traditional Chinese Medicine (TCM) treats various complex diseases (multi-etiologies and multi-symptoms) via herbs interactions to exert curative efficacy with less adverse effects. However, the ancient Chinese compatibility theory of herbs formula still remains ambiguous. Presently, this combination principle is dissected through a systems pharmacology study on the mechanism of action of a representative TCM formula, *Huo-xiang-zheng-qi* (HXZQ) prescription, on the treatment of functional dyspepsia (FD), a chronic or recurrent clinical disorder of digestive system, as typical gastrointestinal (GI) diseases which burden human physical and mental health heavily and widely. In approach, a systems pharmacology platform which incorporates the pharmacokinetic and pharmaco-dynamics evaluation, target fishing and network pharmacological analyses is employed. As a result, 132 chemicals and 48 proteins are identified as active compounds and FD-related targets, and the mechanism of HXZQ formula for the treatment of GI diseases is based on its three function modules of anti-inflammation, immune protection and gastrointestinal motility regulation mainly through four, i.e., PIK-AKT, JAK-STAT, Toll-like as well as Calcium signaling pathways. In addition, HXZQ formula conforms to the ancient compatibility rule of “*Jun-Chen-Zuo-Shi*” due to the different, while cooperative roles that herbs possess, specifically, the direct FD curative effects of *GHX* (serving as *Jun* drug), the anti-bacterial efficacy and major accompanying symptoms-reliving bioactivities of *ZS* and *BZ* (as *Chen*), the detoxication and ADME regulation capacities of *GC* (as *Shi*), as well as the minor symptoms-treating efficacy of the rest 7 herbs (as *Zuo*). This work not only provides an insight of the therapeutic mechanism of TCMs on treating GI diseases from a multi-scale perspective, but also may offer an efficient way for drug discovery and development from herbal medicine as complementary drugs.

## Introduction

It is well known that many complex diseases including CVDs, cancers, HIV, etc., are usually, in character, caused by a combined action of multi-factors (organs, tissues and proteins). Therefore, monotherapies may not always produce ideal efficacy. Whereas, TCM, characterized by “multi-components” and “multi-targets” features and regarded as a precious treasure for Asians, has been applied in treating various complex diseases as principle or auxiliary drugs for more than 2,500 years (Pei et al., 2016). Compared with monotherapies, TCM takes into account most aspects of complex diseases (multi-etiologies and multi-symptoms), and thus often exerts potent curative efficacy. Specifically, in two ways, TCM contributes to the process of turning dysfunctional living organisms back to their normal states: (1) containing plentiful active components which usually provide patients with beneficial synergistic actions by acting on diverse biological targets; (2) being mostly natural herbal medicines, and sometimes even edible, and thus may be of less side effects and low toxicities (Li et al., 2014). Nevertheless, for most TCMs, not only their mechanism of action, but also the compatibility theory their herbs follow still remain vague.

Gastrointestinal (GI) diseases, a kind of highly prevalent complex diseases, account for substantial morbidity, mortality and health care utilization of humankind world (Peery et al., 2015). Since FD, one of the most common diseases of digestive system, is a typical GI disease due to the multi-pathological causes, presently it is used as an example to explore corresponding mechanism involved in the GI diseases therapy. In fact, FD is defined as chronic or recurrent clinical syndrome of upper abdominal with complex pathogenesis, with 7 ∼ 45% current morbidity (Buzas, 2007) worldwide. Its symptoms include epigastric pain or burning, early satiety, belching, nausea, bloating, vomiting, fullness after meal, which are usually attributed to slow gastric emptying, failing of the gastric fundus, visceral hypersensitivity to distention, gastroenteritis, duodenal inflammation, or center nervous system dysfunction (Xiao et al., 2012). HXZQ, a famous TCM recipe described in *Prescriptions of Peaceful Benevolent Dispensary*, has been used for the treatment of gastrointestinal disorder from ancient Song Dynasty in China. The formula is composed of 11 herbs: *Pogostemon cablin* (*Blanco*) *Benth* (*GHX*), *Atractylodes macrocephala Koidz* (*BS*), *Magnolia officinalis Cortex* (*HP*), *Arum ternatum Thunb* (*BX*), *Perilla frutescens* (*ZS*), *A. dahurica* (*Fisch.*) *Benth. Et Hook* (*BZ*), *Citrus reticulata* (*CP*), *Poria cocos* (*Schw.*) *Wolf* (*FL*), *Licorice* (*GC*), *Areca catechu L* (*DFP*), *Zingiber officinale Roscoe* (*SJ*). Although its efficacy in FD treatment has been confirmed by numerous clinical appliances, its fundamental molecular action mechanisms as well as the combination principle of the herbs are still elusive. Thus, taking HXZQ formula for FD treatment as probe, the present work aims at interpreting the compatibility theory and the action mechanism of TCMs in the treatment of GI diseases. Specifically, by applying a systems pharmacology platform, we explore the pathogenesis of FD disease as well as the therapeutic mechanism of HXZQ prescription. The obtained results, we hope, may not only improve the comprehension of FD pathogenesis and HXZQ pharmacological basis, but also promote the development of TCM herbs as complementary drugs for curing complex diseases.

## Materials and Methods

### Database Building

The ingredients of all herbs in HXZQ were data-mined from not only relevant databases including TCM Systems Pharmacology Database (TCMSP^1^), Chinese Academy of Sciences Chemistry Database^2^, Herbal Ingredients’ Targets Database (HIT), TCM database @Taiwan^3^, and TCMID^4^, but also all related literatures. Finally, 1,192 chemicals were obtained with structures collected from NCBI PubChem Database^5^. All structures of these chemicals were drawn and optimized by Sybyl 6.9. HXZQ herbs’ name, the number of ingredients they contain and corresponding abbreviations are shown in Table 1.

**Table 1:** The herbs of HXZQ formula.

No.	Name		Number		Abbreviation
			
	Latin	Chinese pinyin	Components	Candidate compounds	
(1)	*Pogostemon cablin (Blanco) Benth*	Guanghuoxiang	94	7	GHX
(2)	*Atractylodes macrocephala Koidz*	Baizhu	55	9	BS
(3)	*Magnolia officinalis Cortex*	Houpu	139	7	HP
(4)	*Arum ternatum Thunb*	Banxia	116	11	BX
(5)	*Perilla frutescens*	Zisu	328	9	ZS
(6)	*A. dahurica (Fisch.) Benth. Et Hook*	Baizhi	223	6	BZ
(7)	*Citrus reticulata*	Chenpi	63	5	CP
(8)	*Poria cocos (Schw.) Wolf*	Fuling	34	14	FL
(9)	*Licorice*	Gancao	280	73	GC
(10)	*Areca catechu L*	Dafupi	16	2	DFP
(11)	*Zingiber officinale Roscoe*	Shengjiang	264	5	SJ


### Workflow of the Systems Pharmacology Approach

**FIGURE 1:**
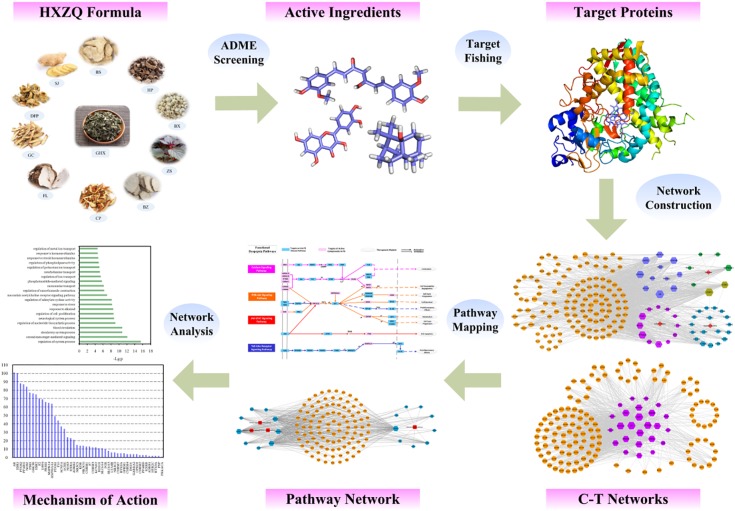
Workflow of the systems pharmacology approach.

The specific workflow is displayed in Figure 1. Firstly, the active components of HXZQ herbs were identified via an ADME-screening model which incorporates the OB, DL and half-life (HL) screening modules together. Then, the potential targets of the prescription were predicted through target fishing with corresponding compound-target networks mapped with attempt to explore the essence of the herbal medicine from a systematic point. Subsequently, target-pathway networks were constructed for further network pharmacology analysis.

### Active Ingredients Screening

#### Oral Bioavailability

As a most vital pharmacokinetic parameter, OB is the rate and percentage of an oral dose for a drug that is absorbed into blood circulation and produces pharmacological effects. Presently, a robust in-house model OBioavail 1.1 was employed to calculate the OB values, which was built based on a dataset composed of 805 diverse drugs or drug-like molecules by consideration of the action of *P*-glycoprotein and Cytochrome P450s in metabolism and information transport (Li et al., 2015a; Wang et al., 2015). Those molecules with OB ≥ 30% were filtered out as candidate compounds with determination coefficient *R*^2^ of 0.80 and SEE of 0.31.

#### Drug-Likeness

Drug-likeness is the comprehensive reflection of a molecule’ s pharmaco dynamics properties in human body, which has been applied in drug discovery to identify those molecules with “drug-like” traits so as to modulate corresponding targets (Wang et al., 2015). Presently, a *Tanimoto* coefficient was applied to calculate the DL value.

$$DL(A,B)=\frac{A*B}{{\left|A\right|}^{2}+{\left|B\right|}^{2}-A*B}$$

in which A represents the molecular descriptors of herbal components, and B the average molecular properties of all 6,511 molecules in DrugBank database^6^ (Wishart, 2006), respectively. In this study, DL ≥ 0.18 is adopted as a filter threshold to screen the active components of herbs.

#### Drug Half-Life

Considering that HL is the essential pharmacokinetic parameter of drugs which represents the time taken for a substance to lose half of its pharmacologic and physiologic activities, we introduce a robust prediction drug half-life model that enables us to forecast long or short half-life of drugs by using the C-partial least square (C-PLS) algorithm (Chung and Keles, 2010), which is supported by 169 drugs with known half-life from DrugBank to acquire potential active ingredients with the screening threshold value defined as HL ≥ 4.

### Target Prediction and Classification

Currently, a computer model established by random forest (RF) and support vector machine (SVM) algorithms which integrates the chemical, genomic, and pharmacological information was applied (Liu et al., 2013) to predict the potential targets with RF score ≥ 0.8 and SVM ≥ 0.7 as threshold. Thereafter, the targets’ information was mined by browsing of the HIT, herapeutic Targets Database (TTD^7^) and DrugBank combined with literatures. All resulted targets were then sent to TTD and PharmGKB^8^ for disease mapping. Finally, the targets were further mapped to UniProt Database^9^ for the normalization of targets’ writing form.

### GO Enrichment Analysis

Presently, the GO enrichment analysis was performed to further probe the vital biological process of achieved targets which were mapped to DAVID (the Database for Annotation, Visualization and Integrated Discovery^10^) for analyzing targets’ biological meaning. The GO terms of biological process were utilized to symbolize genic function. Finally, those GO terms with *p*-value ≤ 0.05 and FDR ≤ 0.05 were selected for further research.

### Network Construction

In this work, two types of biological networks were constructed for HXZQ. Firstly, the compound-target (C-T) networks were generated by Cytoscape v3.2.1 incorporating all the active compounds-targets interactions in HXZQ formula. Then, related pathways obtained by mapping the targets to KEGG database^11^ were used to build compound-target-pathway networks for further network pharmacology analysis (Li and Zhang, 2013; Zhang et al., 2013).

### *In silico* Validation of the C-T Interactions

For exploring the binding modes and offering more insights into the interactions between the candidate compounds and their protein targets, three targets and twelve C-T interactions were selected for docking validations as illustrations. The molecular docking of these protein-ligand complexes was carried out by using GOLD version 5.1, a genetic algorithm-based docking program to generate an ensemble of docked conformations. The X-ray crystal structures of CHRM3, GSK3B and PTGS2 (with PDB entry codes of 4DAJ, 4ACD and 5F19, respectively) were retrieved from RCSB Protein Data Bank^12^. Taking into account the factors including H-bonding energy, van der Waals energy, metal interaction, and ligand torsion strain in the defaulted scoring function, the GOLD Score fitness function was employed.

## Results

### Active Compounds Screening

Since HXZQ formula is composed of 11 herbal medicines with each containing dozens or even hundreds of ingredients, the building of an ingredient database for HXZQ is a necessity. Thus, to our best efforts, by data mining a total of 1,192 molecules were obtained as HXZQ’s components presently.

It is well known that among the great number of compounds contained in a TCM, many chemicals fail in reaching the target sites due to the lack of suitable pharmaceutical properties in oral administration process (Li et al., 2015b). Actually, it is the ADME (absorption, distribution, metabolism, and excretion) properties of a drug that determine its success or failure in this process. Thus, for finding out those possible active ingredients, a screening platform containing three models we established, which, respectively, evaluate three essential pharmacokinetic parameters, namely, OB, DL, and HL, that reflect the most crucial ADME/T properties of compounds, was employed to screen the ingredient database. As a result, candidate compounds which satisfy the conditions of OB ≥ 30%, DL ≥ 0.18, and HL ≥ 4 were sorted out into the candidate compound pool. It is worth noting that several compounds have relatively low pharmacokinetic values, but they are either the richest ingredients of the herbs, like magnolol (HP01) and honokiol (HP02), or biologically active, thus are also considered as candidate components presently. In this way, finally 132 chemicals are identified as active compounds of HXZQ, with information all listed in Supplementary Table S1. Table 2 displays part of them as examples.

**Table 2:** Certain candidate compounds of HXZQ formula mentioned in the present work.

ID	Name	Structure	ID	Name	Structure
BS03	Atractylenolide I		GHX01	Genkwanin	
		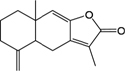			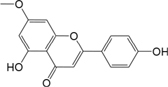
BX02	Cavidine	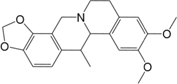	GHX04	Irisolidone	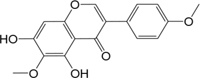
BZ04	Coumarin	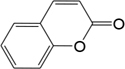	GHX05	Patchouli alcohol	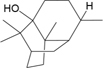
CP03	Nobiletin	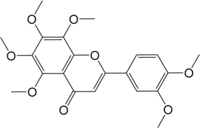	GHX06	Quercetin	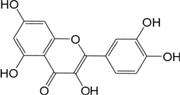
DFP01	Arecoline	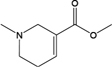	DFP02	Arecolidine	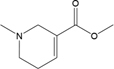
HP01	Magnolol	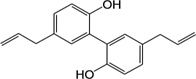	HP02	Honokiol	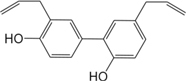
GC35	Licoisoflavone B	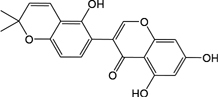	FL10	Pachymic acid	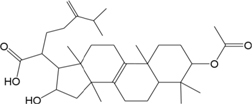
GC11	Medicarpin	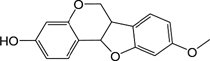	BX03	Baicalein	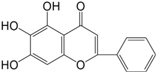
GHX07	Rutin	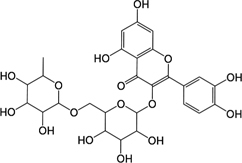	GC69	Glycyrrhizic acid	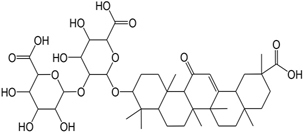
GC13	7-Methoxy-2-methoxyisoflavone	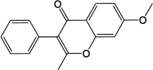	GC08	Inermine	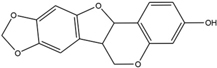
BS0 4	Atractylenolide II	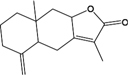	SJ02	Curcumin	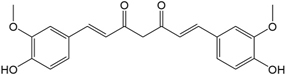


Interesting, among them, plenty compounds have been reported biological active. For instance, baicalein (BX03), a widely reported flavonoid of *BX*, restrained lipopolysaccharide (LPS) -induced NO production which reflects the severity of inflammation (Chen et al., 2001). It also affected the inflammation related cyclooxygenase activities to relieve enterogastritis (Sekiya and Okkuda, 1982). Moreover, BX03 possesses anti-*Helicobacter pylori* (*H. pylori*) activity and thus protected the gastrointestinal digestive tract of FD patients (Bae et al., 1999). Beta-sitosterol is not only the mutual ingredient of five herbs including *HP*, *BX*, *ZS*, *FL*, and *SJ* in HXZQ formula, but also usually used in modulation of immune system, as well as the prevention of cancer or heart diseases (Saeidnia et al., 2014). And its pharmacological efficacy for FD treatment such as anti-inflammatory, analgesic and anthelmintic activities have already been experimentally proved (Saeidnia et al., 2014).

**FIGURE 2:**
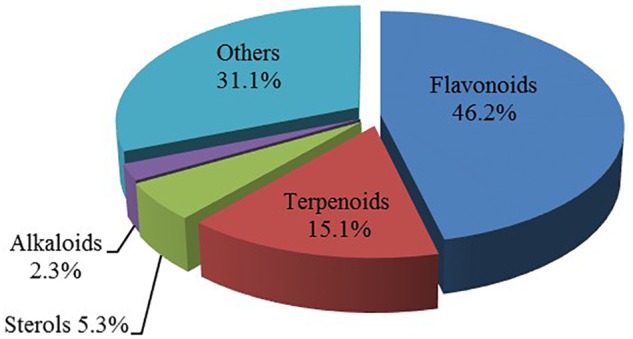
The species distribution of the bioactive chemical pool of HZXQ.

In observation of the structures of the candidate compounds, an interesting phenomenon attracts our attention that many of them lie in two types, i.e., flavonoids and terpenoids. Actually, nearly half are flavonoids, and 15.1% are terpenoids as shown in Figure 2. In nature, flavonoids are widely accumulated in medicinal plants. They are important for plant development, and are also well known as beneficial for human nutrition, health and prevention of cell aging (Hichri et al., 2011). According to structure, the 61 flavonoid active ingredients in HXZQ belong to two categories: 2-phenylchromans (40) and 3-phenylchromans (21, also called as isoflavoids), with either having been reported with a wide range of proper biological activities, including antibacterial, antithrombotic and anti-inflammatory effects (Knekt et al., 2002). For example, quercetin (GHX06), a typical flavonoid in plants, exerts remarkable antioxidative and anti-inflammatory effects and thus is capable of treating gastrointestinal inflammation and relieving FD patient’s pain. Factually, GHX06 up-regulated several pro-inflammatory mediators like TNF-α and IL-1β in model rats, and in this way fulfilled its anti-inflammatory functions (Ji et al., 2017). GHX06 has also analgesic effects through inhibiting the nociceptive neurotransmission and ameliorating the pathological pain. Specifically, a continuous daily administration of GHX06 at 100 mg/kg for 14 days attenuated the hyperalgesia of model rats in the long-term pain treatment trails (Ji et al., 2017). In addition, licoisoflavone B (GC35), an isoflavone compound, reduced the damage of the gastrointestinal mucosa caused by *H. pylori*, by potently inhibiting the growth of *H. pylori* ATCC 43504, ATCC 43526, ZLM 1007, and GP98 even with a minimum inhibitory concentration of 6.25 μg/mL *in vitro* (Fukai et al., 2002). Therefore, flavonoids, the main ingredients of HXZQ, should be the major molecular bioactivities basis of this formula.

In addition, terpenoids, another major category of HXZQ’s active components, are defined as the derivative of mevalonic acid conforming to the (C_5_H_8_)_n_ general structure. Terpenoids play essential roles in the basic life of plants, and are also applied in industrial production and medical hygiene. For instance, atractylenolide I (BS03), a sesquiterpene derived from herb *BS*, possesses neuroprotective, all-allergic, anti-inflammatory and anticancer bioactivities (Fu et al., 2018). For inflammatory model mouse, the treatment of BS03 factually significantly decreased the levels of pro-inflammatory factors TNF-α and IL-6 in a dose-dependent manner (Wang et al., 2016). Thus, terpenoids are also important active substance of HXZQ.

Besides, HXZQ also contains some other kinds of chemicals like sterols and alkaloids. Actually, many plant sterols have inhibitory functions for the growth of tumors (Rubis et al., 2008). Similarly, alkaloids are also one kind of effective components of TCM. Arecoline (DFP01), the main active constituent of *DFP*, promotes intestinal peristalsis and enhances GI motion. Experiments showed that DFP01 enhanced bowels’ tension in rabbits through mediating muscarinic acetylcholine receptor (Xie et al., 2004). Other categories of substances like essential oils and organic acids in HXZQ may provide nutrition or a proper microenvironment *in vivo*.

In summary, due to diversified structural distribution of its substance basis, i.e., the various types of candidate compounds that cover flavonoids, terpenoids, sterols and alkaloids etc., HXZQ is capable of holistically treating the complex etiology of FD from several different aspects.

### Physicochemical Property Analysis

In order to analyze the drug-like physicochemical properties of active compounds, a comparison of the properties of the herbal active ingredients and DrugBank medicines is carried out by consideration of eight common molecular descriptors, which include MW (molecular weight), nCIC (number of rings), nHDon (number of hydrogen-bond donors), nHAcc (number of hydrogen-bond acceptors), RBN (number of rotatable bonds), Hy (hydrophilic factor), TPSA (topological polar surface area) and MlogP (Moriguchi octanol-water partition coefficient), since that these parameters reflect the basic characteristics of the molecules including especially their pharmacokinetic properties (Li et al., 2017).

**FIGURE 3:**
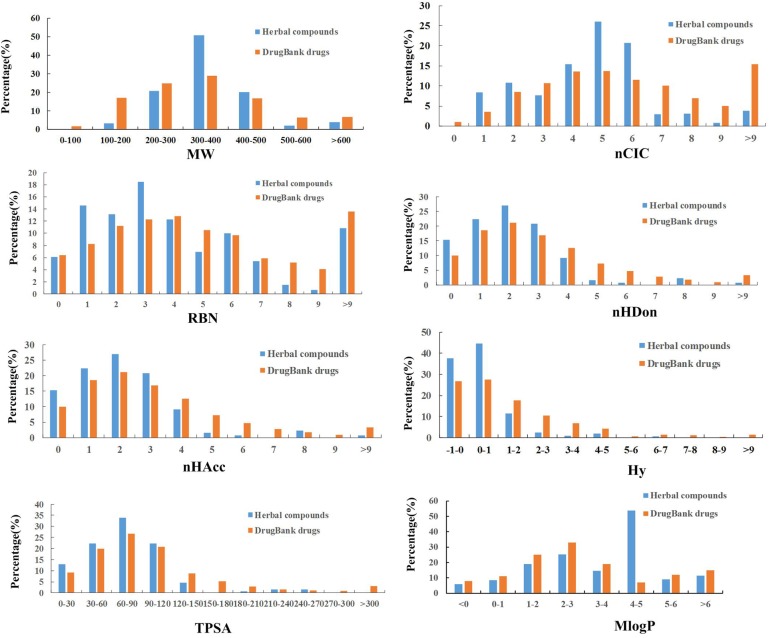
The profile distributions of eight important molecular properties of DrugBank drugs and herbal compounds.

Lipinski’s rule of five is a rule of thumb to evaluate the DL or determine if a compound with a certain pharmacological or biological activity has chemical and physical properties to make it a likely orally active drug in humans. The specific content of this rule is, in general, an orally active drug has no more than one violation of the following criteria: possessing (1) no more than 5 hydrogen bond donors; (2) no more than 10 hydrogen bond acceptors; (3) a molecular weight less than 500 daltons; (4) an octanol-water partition coefficient logP not greater than 5 (Lipinski et al., 2001). As seen from Figure 3, four parameters including nHDon, nHAcc, MW, and MlogP are all related to this principle and meet the rule well, indicating that the active ingredients of HXZQ formula are very likely to become drugs. In addition, concerning with the molecular weight, herbal chemicals and DrugBank compounds have quite similar tendency (with *p* > 0.05) based on the factor analysis of variance that they both follow a Gaussian distribution characteristics. Whereas, significant difference exists between the herbal and DrugBank chemicals in nHDon, nHAcc, and MlogP as shown in Table 3 (with *p* < 0.01). Specifically, for nHDon and nHAcc, the average values of HXZQ formula are lower than DrugBank, which illustrates that the average number of hydrogen bonds generated by HXZQ molecules may be less than DrugBank. As to MlogP, the average value of HXZQ’s active compounds is larger than that of DrugBank, implying that the active ingredients of herbs probably are more lipophilic and more soluble in the lipid solution. Actually, this corresponds well with another index, Hy (in Table 3), that Hy’s average value of HXZQ formula is lower than the DrugBank one, suggesting that the herbs have fewer hydrophilic molecules and lower overall hydrophilicity.

**Table 3:** Comparison of the molecular properties between herbal and DrugBank compounds.

Index	MW (±*SD*)	nCIC (±*SD*)	RBN (±*SD*)	nHDon (±*SD*)	nHAcc (±*SD*)	Hy (±*SD*)	TPSA (Tot) (±*SD*)	MlogP (±*SD*)
**DrugBank**	346.57 (208.89)	2.46 (1.72)	5.58 (5.88)	3.17 (3.50)	6.46 (5.59)	1.47 (2.76)	99.93 (90.43)	1.33 (2.50)
**Herbal**	358.31 (103.34)	3.11 (1.37)^∗∗^	4.83 (5.68)	2.14 (1.73^)∗∗^	4.79 (2.67)^∗∗^	0.40 (1.19)^∗∗^	76.55 (43.93)^∗∗^	2.96 (2.14)^∗∗^


*^∗∗^p < 0.01.*

Among the other three indices that are also displayed in Table 3, namely, nCIC, TPSA, and RBN, some difference is observed for the former two parameters that the average value of TPSA is lower than those of DrugBank ones, indicating that the compounds in herbal formula are more likely to permeate the membrane and be absorbed by human body. Whereas, for nCIC, its average value of herbal compounds is larger than those drugs in DrugBank database, indicating that HXZQ formula contains many aromatic compounds. As to RBN, little difference exists between herbal and DrugBank database, implying similar flexibility of herbal compounds to DrugBank ones. In a word, the herbal chemicals are characterized by large number of aromatic components, relative high hydrophobicity and moderate molecular weight, and therefore may be easily absorbed by human body.

### The Combining Rule of “*Jun-Chen-Zuo-Shi*”

**FIGURE 4:**
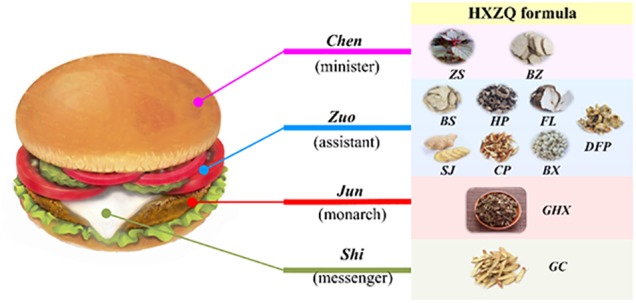
The combination rules of HXZQ herbs based on the TCM “*Jun-Chen-Zuo-Shi*” theory.

It is well known that the formation of any TCM formula composed of multiple or dozens of herbs is not random. Instead, almost all of them are built based on certain combinational rules, among which the most famous one is the ancient Chinese theory of “*Jun-Chen-Zuo-Shi*”. Its basic assumption holds that if a TCM prescription functions like a government, then its herbs should fulfill different duties including *Jun* (monarch), *Chen* (minister), *Zuo* (assistant) and *Shi* (guide) either individually or in a combinational way to ensure the smooth run of the formula. Presently, to vividly depict the theory, a salutary meat burger is drawn (as shown in Figure 4), where the meat, bread, vegetables and cream correspond to *Jun*, *Chen*, *Zuo*, and *Shi*, respectively, according to the different roles they play individually if the burger is considered as a TCM formula.

As is known to us, all FD pathological factors belong to three classes, i.e., the gastrointestinal damage caused by bacterial (including especially *H. pylori*) infection, the inflammation of gastrointestinal tract, and the inadequate gastrointestinal motility in essence. Therefore, presently, we first analyzed the therapeutic functions of individual herbs of the formula on FD treatment from these three classes of factors. Then, based on the analysis of the structural distribution of active compounds in the herbs and the contribution of their specific activities to FD treatment, as well as the investigation and understanding of traditional combination theory about the roles of the main and auxiliary drugs of TCM, we conclude that HXZQ formula conforms to the compatibility rule of “*Jun-Chen-Zuo-Shi*,” which theory can be scientifically interpreted as herbs playing different roles based on their specific contributions to the integrated therapeutic function of the TCM formula. In other words, *Jun* (monarch) drug, as the most essential herb/herbs in a TCM prescription, plays lead pharmacological activities in prevention and treatment of disease as the enlightened monarch in a powerful nation. *Chen* drug, like a minister, either promotes the curative effects of *Jun* drug or is responsible for treating some accompanying symptoms (Wu et al., 2014). *Zuo* (assistant) drug enhances the minister drug’s treatment efficacy, or aims at minor symptoms. As for *Shi* drug, it is generally used in low dose, with aim to induce the herbs’ impact to the disease location and modulate the interaction among the herbs, as well as to remove toxic substance from the human body, which is also called as a “messenger” drug. This principle signifies the fact that each herb of the recipe has a specific function within the composition, and is organized and arranged integrally under the principle to generate specific effects.

Presently, during the revision process, based on this assumption and the regular dosage of the herbs of HXZQ formula, the influence of the quantity of all medicines in the formula was also investigated. Specifically, in general the dosage of *GHX* and all other herbs follows a fixed ratio (as shown in Table 4), where the content of *SJ* is usually ignored due to the fact that *SJ* is only used as soaking solution of *HP* during the preparation process of the herbs before all processed herbs come together as a formula. Thus, based on this dose ratio, the relative blood concentrations of all herbs in HXZQ formula are calculated, which ends up with results as shown in Table 4.

**Table 4:** The number of molecules and relative blood concentration of each herbs.

				Relative blood
	Number of		Dose	concentration
Herb	ingredients	OB ≥ 30% (%)	ratio	(OB × dose ratio/35) (%)
*GHX*	94	60.6	6	10.4
*DFP*	16	43.8	2	2.5
*BZ*	223	48.4	2	2.7
*ZS*	328	51.8	2	3.0
*FL*	34	52.9	2	3.0
*BX*	116	53.4	4	6.1
*BS*	55	21.8	4	2.5
*CP*	63	57.1	4	6.5
*HP*	139	61.1	4	7.0
*GC*	280	51.1	5	7.3


*GHX*, belonging to Lamiaceae family, is the dried aerial part of a famous herb named “*Guanghuoxiang*.” Actually, it is a frequently used folk medicine in the treatment of common cold, nausea, diarrhea, rhinitis, headaches and fever, and it bilaterally regulates the gastrointestinal smooth muscle, accelerates the secretion of digestive juice, and protects the intestinal barrier function. Clinically, *GHX* has been long used as a major composition of HXZQ formula due to its multiple beneficial biological activities such as the anti-inflammatory, anti-fungal and gastrointestinal tract regulation effects (Singh and Ganesha Rao, 2009). Presently, *GHX* contains 94 ingredients, where 7 compounds are identified as candidate compounds. Although *GHX* has only limited number of active ingredients, most of them directly contribute to the therapy of GI diseases. For example, irisolidone (GHX04), a major isoflavone in *GHX*, has a wide spectrum of favorable bioactivities such as antioxidative, antiviral, anti-inflammatory and anti-tumor (Park et al., 2006; Kang et al., 2008). It also inhibits the production of LPS-induced NO, cytokines TNF-α to relieve enterogastritis and the expression of matrix metallo proteinases which control the tumor invasion and angiogenesis (Kim and Yun-Choi, 2008). In addition, patchouli alcohol (GHX05), *GHX*’s principal ingredient and a tricyclic sesquiterpene, also demonstrates high selective antibacterial effects against *H. pylori* (Liao et al., 2013). As a matter of fact, GHX05 potently inhibits the inflammatory response through decreasing those inflammatory mediators including TNF-α, IL-1β, and NO in LPS-stimulated RAW264.7 macrophages (Li X. et al., 2012; Li Y. et al., 2012). As seen from Tables 5, 4, the relative blood concentration of *GHX* is far higher than all other herbs, which, combined with the broad antibacterial activities as well as the good anti-inflammatory activity the herb possesses, verifies the *Jun* role of *GHX* in HXZQ formula. Besides, *GHX* is the most abundant herb in HXZQ, which may also account for its prominent roles in the formula.

**Table 5:** The content, OB and blood concentration of three chemicals of *GHX.*

			Blood concentration
Chemical name	Content (mg/g)	OB (%)	(mg ⋅ %/g)
Isoverbascoside	3.101	2.94	0.091
Pogostone	1.445	30.17	0.430
Cernatoside	0.732	2.74	0.020


*ZS* and *BZ* are *Chen* drugs of HXZQ. As mentioned earlier, *Chen* drugs exert their medicinal properties from two aspects: (1) enhancing curative effect of *Jun* drug; and/or (2) treating some accompanying symptoms. *ZS* is a traditional herb with a specific aroma which is in charge of the treatment of bacterial and fungal infections. Interestingly, this plant is not only medicable, but also an edible spice. Studies have shown that those components exerting the main effects of *ZS* are rosemary acid and perillyl alcohol (Gu et al., 2009). Whereas, perillyl alcohol possesses excellent anti-cancer effects which has been applied clinically currently (Loutrari, 2004). Although its bioactivities for GI system have not been reported, perillyl alcohol is assumed to have potentials for FD treatment and is worthy of further research and exploration. As for *BZ*, it has strong antipyretic, analgesic, antiasthmatic, antispasmodic and antibiosis effects, which are also part of major accompanying symptoms of FD. In fact, one BZ active ingredient, coumarin (BZ04), not only remedied the pain caused by glacial acetic acid in rats and intestinal smooth muscle spasm caused by BaCl_2_ in rabbits, but also exhibited anti-inflammatory effects (Zheng et al., 2010). In short, despite of the relatively low levels of relative blood concentration, *ZS* and *BZ* are not only responsible for producing direct curative effects by bactericidal action, but also in charge of treating certain accompanying symptoms (like gastrospasm and pain) of FD, and thus serve as *Chen* drugs in HXZQ formula.

It is well known that *Zuo* drugs usually function either by treating the minor accompanying symptoms or improving the efficacy of *Chen* drugs in a TCM prescription. Thus, 7 herbs including *BX, HP, FL, SJ, CP, BS*, and *DFP*, attract our attention due to their similar functions in HXZQ formula. In fact, in FD therapy, these drugs mainly undertake a function of promoting the insufficient gastrointestinal motility and dealing with certain minor symptoms like abdominal distension, belching, nausea and vomiting. For example, *HP* has been extensively applied in the treatment of abdominal distention, pain and dyspepsia in Asia for long time (Yu et al., 2012). Some reports highlight that *HP*’s extract may protect central nervous systems and exhibit anxiolytic effects (Xu et al., 2008). As seen in Table 1, *HP* contains 7 active compounds, from which five molecules including eucalyptol, beta-sitosterol, neohesperidin, HP01 and HP02 exhibit prominent biological activities. Eucalyptol, a saturated monoterpene, exerts various bioactivities including inhibiting cyclooxygenase pathway, suppressing the arachidonic acid metabolism or cytokine production, as well as proper anti-inflammatory effects in rats (Juergens et al., 2003, 1998). Beta-sitosterol, another potent bioactive molecule, widely distributes in various botanicals with blood cholesterol lowering effects reported (Lee et al., 2007). As for neohesperidin, it produces significant antioxidant activities when relieving gastric lesions, and increases the mucus content. In addition, it has also protective effects by significantly decreasing the volume of gastric secretion to prevent gastric dysfunction (Lee et al., 2009). Two other parallel prime bioactive constituents of *HP*, HP01 and HP02, are both isomers of hydroxylated biphenolic compounds, and both relieve the spasm of smooth muscle and vomiting. Actually, they have demonstrated anti-diarrhea effects by blocking the calcium channel to inhibit the abnormal intestinal ion transport (Park et al., 2004), as well as improving the gastric emptying and intestinal propulsive actions (Zhang et al., 2005). Therefore, these seven herbs with moderate relative blood concentration as demonstrated, we assume, serve as *Zuo* drugs of HXZQ formula mostly by treating those minor symptoms of FD disease.

Although exerting certain beneficial bioactivities, some alkaloids, like arecoline and arecolidine, still have side effects. Whereas, *GC* has certain detoxification function like that its unique ingredient glycyrrhizic acid (GC69) reacts with these alkaloids and hence weakens their adverse effects. Thus *GC* is widely used in concerted application of botanical drugs as a crucial *Shi* drug. In fact, out of all current TCM prescriptions, about 60% contain *GC*, thus this herb is almost the most typical *Shi* drug of herbal formulae. In addition, *GC* also regulates CYP450 enzymes which are primary phase I isoenzymes in liver responsible for the metabolism of almost all drugs and toxins and hence influences the metabolism property of other herbs. For instance, its ingredient GC69 interacts with CYP3A4 in enterocytes which results in a significant activation of the functions of CYP3A4 (Hou et al., 2012). Besides, in *Chai-huo-shu-gan-san* formula, *GC* significantly increases the release of Bupleurum, the formula’s *Jun* drug, and in this way promotes the efficacy of the formula. Besides, *GC* also regulates the function of certain transporters like *p*-glycoprotein which is an important protein of the cell membrane that pumps many foreign substances (drugs) out of cells and therefore systematically impacting the delivery of most drugs to their targets. Additionally, due to the wide spectrum of targets GC possesses, which are widely distributed in almost all vital organs including the cardiovascular, respiratory, GI and nervous systems, *GC* pharmacologically influences, basically, the whole human body (Liu et al., 2013). In fact, *GC* possesses a broad range of activities including antiviral, anti-inflammatory, anti-tumor, immunostimulant, anti-oxidant, antispasmodic metabolic syndrome prevention activities. Moreover, *GC* also contains some common ingredients with other herbs like GHX06, CP01, which may produce cross-interactions with other herbs’ chemicals, and in this way regulate the relationships among herbs. *GC*, on one hand, has relatively high relative blood concentration (as shown in Table 5). On the other hand, it also exerts multiple biological functions, including (1) the detoxication capacity to reduce side effects that other herbs may produce, (2) large number of structural diverse active compounds, and (3) broad spectrum of bioactivities which are involved in the regulation of the ADME properties of other herbs. By consideration of all these factors, *GC* is assumed as *Shi* drug to coordinate other herbs as well as an antidote agent in HXZQ formula.

### Target Identification and Network Pharmacology Analysis

Presently, for HXZQ formula, altogether 48 proteins are identified as its targets, with Supplementary Table S2 listing all corresponding detailed information. Interestingly, the association of many of them with FD treatment has been validated, like dipeptidyl peptidase 4 (DPP4), nitric oxide synthase, prostaglandin G/H synthase (PTGS2), Glycogen synthase kinase 3 beta (GSK3B) (Gao et al., 2014). Thus, based on these targets and corresponding interacting compounds, compound-target (C-T) networks are established for HXZQ formula presently, with network pharmacology analysis conducted for analyzing the interaction mechanism of HXZQ-FD complex system.

#### C-T Network and Analysis

**FIGURE 5:**
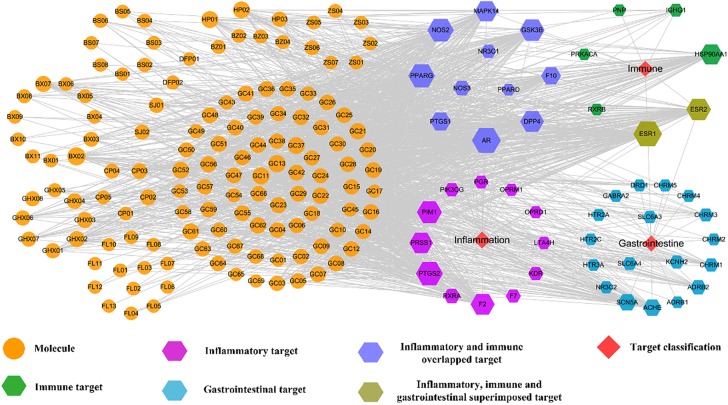
C-T network of the HXZQ formula, where circles and hexagons represent bioactive compounds and corresponding FD targets, respectively. Among the targets, purple, green and blue hexagons represent the inflammatory, immunological and gastrointestinal targets, respectively. The blue violet hexagons are immune and inflammation overlapped targets, whereas the olivaceous hexagons are the immune, inflammation and gastrointestine superimposed targets, respectively. Node size is proportional to its degree.

Firstly, a C-T network is constructed by using all 132 active components of HXZQ and their corresponding 48 targets, which is shown in Figure 5 where the circles and hexagons represent the candidate compounds and targets, respectively.

In this work, to quantify the influence of the nodes and to identify the most influential ones within a network, two important parameters, i.e., degree, which is the number of edges connected to the node, and betweenness, another centrality index defined by the number of times a node acts as a bridge along the shortest path between two other nodes, were calculated. Actually, betweenness reflects the fraction of the shortest paths in the network that pass through any particular node and a measure of the importance of a node as a hub in a network (Grobelny et al., 2018). This measurement favors the nodes that act as connecting links between dense subnetworks, rather than nodes that lie inside a subnetwork. Therefore, if the degree values of some targets or molecules are insignificant but their BCs have relatively high values, these targets or active compounds are also important for the net.

**FIGURE 6:**
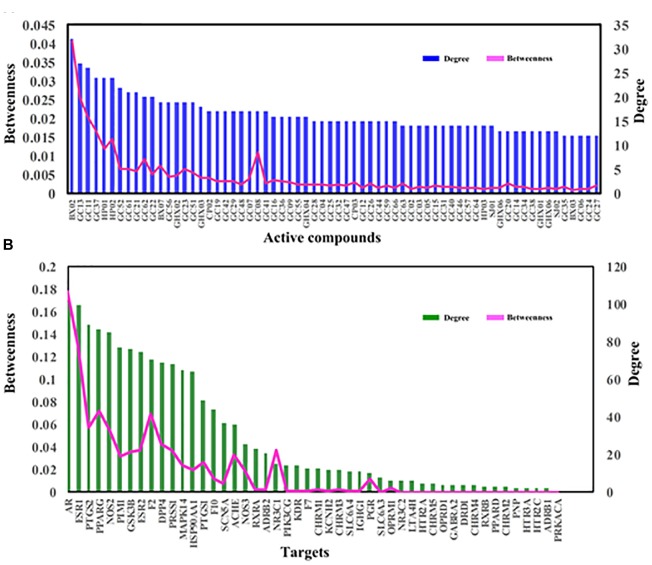
The degree and betweenness of active compounds **(A)** and targets **(B)** of HXZQ formula.

Figure 6 displays the degree and betweenness distribution of all targets and top 60 active compounds. As seen from the figure, in general, the higher degree, the higher betweenness. And the distribution of degree and betweenness is strongly correlated with each other and the most highly connected nodes have higher centrality scores. Still, several nodes are also noticed that they possess high betweenness values despite of relatively low degrees, which may be due to that they connect certain high-degreed nodes. Thus, those nodes at the peaks of the betweenness line, whether as targets (like ACHE, NOS3, NR3C1 and PGR) or candidate compounds (like GC08), are also important for the formula.

Among the targets, the top three degree-ranked proteins are androgen receptor (AR,), estrogen receptor α (ESR1) and prostaglandin G/H synthase 2 (PTGS2), connecting with 101, 100, and 88 compounds, respectively (Figure 6B), indicating their essential roles for the network. As to the average degree of all targets, it is as big as 30, and, actually, the degree values of 17 out of all 48 targets are larger than this mean value. These facts all prove that HXZQ formula exerts its efficacy through a multi-target cooperative mechanism.

As to candidate compounds, cavidine (BX02), 7-methoxy-2-methyl isoflavone (GC13) and medicarpin (GC11) are the top three ones, interacting with 32, 27, and 26 targets, respectively. Actually, their crucial bioactivities for remedying FD have been experimental validated. For instance, BX02 is an isoquinoline alkaloid that has wide spectrum of biological activities including anti-tumor, anti-bacteria and especially anti-inflammation effects (Niu et al., 2015). Actually, BX02 not only decreases the expression of various inflammatory mediators such as nitric oxide (NO), PGE_2_ and cytokines like TNF-α and interleukin (IL-6), but also exhibits relative low cytotoxicity (Niu et al., 2015). Thus, these highly connected chemicals are key to HXZQ for exerting proper efficacy. Besides, the average degree of candidate compounds is also as big as 10.7, proving the multi-ingredient cooperative mechanism of the formula.

**FIGURE 7:**
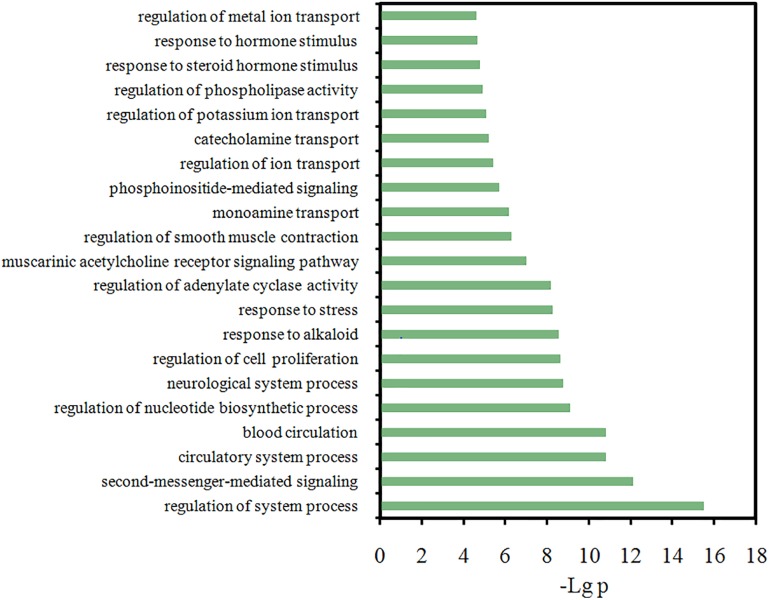
Gene Ontology (GO) analysis of the target genes, where *y*-axis is the significantly enriched ‘Biological Process’ categories in GO relative to the target genes, and *x*-axis is the enrichment scores of these terms (*p*-value ≤ 0.05 and FDR ≤ 0.05).

Actually, to explore which microscopic biological processes these targets are involved in, presently a GO analysis was further performed. Figure 7 displays the most significantly enriched GO terms, with their *p*-value and FDR shown in Supplementary Table S3. Actually, a majority of the targets are closely related to several or more biological processes, such as the regulation of second messenger-mediated signaling, neurological system process, the response to hormone stimulus and the regulation of smooth muscle contraction. And most biological processes among the listed terms are related to FD pathogenesis. In addition, interestingly and also similar to our above results, majority of these highly enriched GO terms are found tightly associated with the inflammation, immune and gastrointestinal systems (Figure 7), like that the “muscarinic acetylcholine receptor signaling” and “regulation of smooth muscle contraction” are closely related to GI motility, and “response to hormone stimulus” is associated with inflammation and immune response. This corresponds well to the experimental findings that FD is conventionally caused by three primarily physiology reasons, i.e., bacterial infection, gastroenteritis and the disturbance of gastric physiologic factors (Talley and Ford, 2015). Therefore, to deeply explore the interaction mechanism of HXZQ formula for FD treatment, currently three C-T networks, i.e., Inf-C-T (Inflammation-Compound-Target), Imm-C-T (Immune-Compound-Target) and Gas-C-T (Gastrointestine-Compound-Target) networks were built from these three pathogenic factors.

##### The anti-inflammation function

**FIGURE 8:**
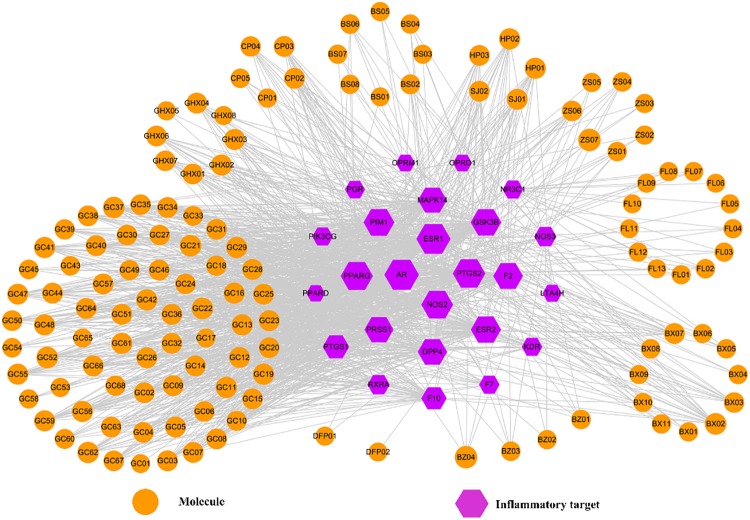
The Inf-C-T network of HXZQ formula, where hexagons and circles represent all inflammation-related targets and corresponding candidate compounds, respectively. The gray edges represent the mutual relation among the targets and chemicals. Node size is proportional to its degree.

It is well known that a typical symptom of FD is the inflammation of GI tract, i.e., gastroenteritis, which is a common disease characterized by diarrhea, vomiting, abdominal pain and fever. Factually, gastroenteritis is also an essential cause for the formation and development of FD. From ancient beginnings, HXZQ has been found exerting significant curative efficacy on gastroenteritis as a standard finished drug. And this efficacy is obtained mostly due to the formula’s anti-inflammatory effects which have already been verified in many studies, like by the decreased levels of TNF-α in peripheral blood and enteric tissue homogenates of the lab mice that were treated with HXZQ (He et al., 2006). All of this arouses our interest to investigate the mechanism of HXZQ’s anti-inflammatory function. Thus, an Inf-C-T network was constructed presently by using all 25 inflammatory targets and corresponding interacting compounds of HXZQ, where the hexagons and circles represent the targets and active ingredients, respectively (Figure 8). Interestingly, the calculated average degree of these inflammatory targets is 48, larger than the average degree of all targets of the formula (30), indicating that anti-inflammatory function may account for the main curative effects of the formula for FD treatment.

Since PTGS1 and PTGS2, two isoforms of cyclooxygenase, are the anti-inflammation targets of most NSAIDs (non-steroidal anti-inflammatory drugs like aspirin and ibuprofen), a kind of currently widely used typical anti-inflammatory medicine, they are firstly investigated in the Inf-C-T network. In fact, their anti-inflammatory function is closely related to the production of PGE_2_, a family member of eicosanoids and also a lipid regulator (Langenbach et al., 1995). PGE_2_ is in charge of the maintenance of the normal blood flow of gastric tissue, and the inhibition of H^+^ formation to protect gastric mucosa. PGE_2_ not only participates in the regulation of different stages of inflammatory response, but also is critical for the maintenance of the health of gastric mucosa (Parente and Perretti, 2003). Whereas, PTGS1 and PTGS2 enhance PGE_2_’s level, and thus their inhibition always leads to the inhibition of PGE2’s production, which finally results in anti-inflammation effects.

Though both possessing similar anti-inflammation bioactivities, in expression, PTGS1 and PTGS2 differ a lot. For PTGS1, it is basically constitutively expressed throughout the whole GI tract, and thus detected in almost all types of cells in normal tissue’s inner muscular layer or even rare villous epithelial cells in mucosa. Whereas, PTGS2 is not detectable in normal GI cells, but only expressed in inflammatory cells (Chulada et al., 2000). Hence, PTGS1’s inhibition influences all cells of GI tract; whereas PTGS2’s inhibition only affects inflammatory cells. For example, when inhibiting PTGS1, NSAIDs produce not only anti-inflammatory effect, but also certain side effects like reduced synthesis of prostaglandin, gastric toxicity, ulcer formation or gastric mucosa damage. Nevertheless, the inhibition of PTGS2 only results in preferable anti-inflammatory effects, with no bad impacts on the GI mucosa detected (Williams et al., 1999). Therefore, if the active ingredients of HXZQ largely inhibit PTGS2 instead of PTGS1, the formula may produce less GI toxicity when treating gastrointestinal inflammation.

Though in this network, in degree PTGS2 is larger than PTGS1 (88 vs. 49) implying more compounds in HXZQ targeting PTGS2 than PTGS1, still there are 49 chemicals interacting with PTGS1 which may produce certain GI toxicity. Yet, for HXZQ up to date only mild toxicity is reported, indicating that this side effect has been somehow eliminated. The reason, we assume, is closely related to the mutual interactions among the chemicals and targets of the formula, which may offset and reduce the potential side effects due to their promiscuous properties. For instance, genkwanin (GHX01), as an active component in HXZQ, targets both PTGS1 and PTGS2. GHX01 has a variety of pharmacological effects including anti-bacterial, radical scavenging and anti-inflammation. GHX01 potently decreases the level of proinflammatory mediators, such as iNOS, TNF-α, IL-1β, and IL-6 (Gao et al., 2014). Through interacting with PTGS1, GHX01 may produce certain side effects in GI mucosa, which yet have not been found. The major reason may be that GHX01 also interacts with other targets that are tightly implicated in inflammatory gastrointestine, i.e., PTGS2, PRSS1, GSK3B, MAPK14, PPARG, NOS2, and ESR2. The interactions among these targets and PTGS1, may relieve gastroenteritis and counteract possible adverse effects (Zhang et al., 2015). Factually, during the long time that HXZQ herbs are used for treating gastroenteric disorders in oriental countries, less or no side effects on GI tract are reported.

Two other connected proteins, i.e., GSK3B and PPARG, also attract our attention that we assume their regulation by HXZQ formula should be helpful for controlling the inflammation of FD due to their pivot position in Inf-C-T network, with connection degree of 76 and 87, respectively. Actually, they both exhibit potent anti-inflammatory effects. For instance, GSK3B plays a crucial role in innate and adaptive immune responses in inflammation-mediated disease treatment. Specifically, its inactivation augments the production of anti-inflammatory cytokine production and synchronously suppresses the expression of pro-inflammatory cytokines in immune cells (Wang et al., 2011). As for PPARG, it is a regulator in charge of the lipid metabolism, glucose homeostasis and cellular differentiation predominantly expressed in intestine. Since its activators have anti-inflammatory activities in monocyte/macrophages, endothelial, epithelial and smooth muscle cells, PPARG has been proven beneficial for the treatment of inflammatory GI diseases (Chinetti et al., 2000). And its modulatory function in control of inflammatory progress with therapeutic applications in inflammation-related gastrointestinal upset was also validated (Chinetti et al., 2000).

In addition, other three highly degreed proteins AR (Degree = 101), ESR1 (100) and ESR2 (75) also arouse our attention due to that they are all key steroid receptors *in vivo*. Their crucial roles in regulation of the central nervous system, cardiovascular system and digestive system, as well as the reproductive system have long been well known. From the large number of active ingredients they interact with (Figure 8), it is speculated that they may also be of significance for anti-inflammatory function of the formula. Actually, in GI inflammatory diseases, ER receptors bring favorable anti-inflammation activities by inhibiting the production of inflammatory cytokine, such as NO, IL-1β, TNF-α (Harnish et al., 2004). In addition, AR, a steroid receptor superfamily member, is an important protein for human genital system. Androgen-bound AR functioning as a transcription factor is involved in an array of physiological processes including especially the inflammatory response. Actually, AR exerts anti-inflammation effects may by decrease of the production of pro-inflammatory factors (like IL-β). In a word, steroid receptors like ESRs and AR are key anti-inflammation targets in the treatment of GI disorders.

In short, the anti-inflammatory function of HXZQ accounts for most of its curative effects on FD treatment, which may mainly attribute to the crucial roles of pivot hub proteins including especially the NSAIDs-targeting cyclooxygenases (PTGS1 and PTGS2), GSK3B and PPARG, as well as steroid receptors (AR and ESRs).

##### The immune protection function

**FIGURE 9:**
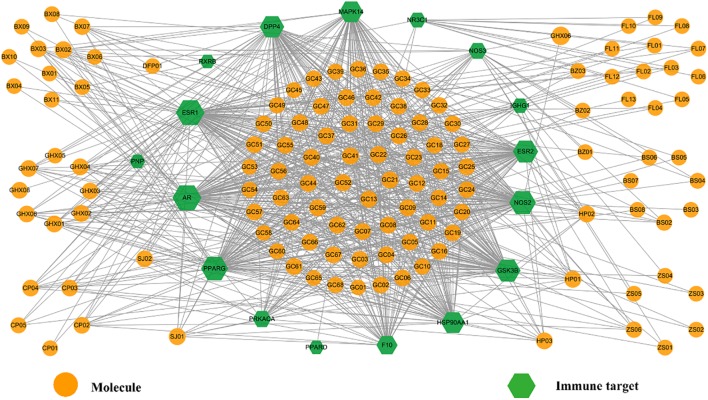
The Imm-C-T network of HXZQ formula, where hexagons and circles represent the immunization-related targets and corresponding active compounds, respectively. The gray edges represent the interaction among the targets and chemicals. Node size is proportional to its degree.

One important cause of FD is the invasion of viruses and bacteria, such as *Salmonella*, *Escherichia coli O157*, *Campylobacter jejuni*, *Giardia lamblia*, *Norovirus*, and *H. pylori*, which always lead to, firstly, mild immune disorders and then gradually FD. Autophagy is induced against all harmful pathogens, among which *H. pylori*, a bacterium capable of adapting to stomach environment and thus living in gastric mucosa, is a typical pathogenic factor of stomach and intestine. Actually, it adheres to mucosal epithelial cells and stimulates the gastric mucosa to produce inflammatory factors and thus leads to a variety of upper GI disorders, such as chronic gastritis, peptic ulcer disease and even gastric cancer (Kusters et al., 2006). Hence, it is worthwhile to explore the impact of HXZQ formula on this germ. Actually, on one hand HXZQ enhances the immunity of patients and inhibits certain bacteria which may induce FD (He et al., 2006). On the other hand, HXZQ also up-regulates CD4^+^ to the normal content range, indicating its roles of repairing the damaged immune system (He et al., 2006). Based on these, presently an Imm-C-T network was built by using all 17 immune-related targets (represented as green hexagons) and related bioactive ingredients (as orange circles) of the herbs (Figure 9), with attempt to explore the influence of HXZQ on immune system.

A major pathological cause of FD is the immune disorder caused by the invasion of harmful bacterium, therefore anti-bacterium is one effective means against GI disorders. In the clearance of invading pathogen, autophagy is an important process in immune response, which is in essence an intracellular degradation system that delivers cytoplasmic constituents to the lysosome (Mizushima, 2007). The autophagy activation is a catabolic process which degrades excrescent and impaired organelles, cytosolic proteins, and invasive microbes. Due to its unique immunological functions, autophagy is involved in many essential processes in the innate and adaptive immune responses (Nys et al., 2013). During the developing process of gastrointestinal disorders, the activation of autophagy in the zoic gastric epithelial is beneficial to gastric epithelial cells (Deretic et al., 2013). Therefore, autophagy is crucial for the inhibition of the growth of various pathogens and the enhancement of human immunity in the treatment of GI diseases.

From Imm-C-T network, it is observed that many active chemicals and targets are involved in autophagy (specifically anti-*H. pylori*) processes, therefore it is presumed that one pharmacological function of HXZQ is the regulation of immune response for FD treatment. Actually, candidate compounds like SJ02, GHX05, GHX01, GHX06, GHX07, HP01, and HP02, all have been proven with inducing autophagy or anti-bacterium activities. For instance, curcumin (SJ02), a representative component of herb *SJ*, is a yellow pigment commonly used in food. It possesses a variety of favorable pharmacological effects like anti-oxidant, anti-inflammatory, hepatoprotective and anti-tumor abilities, which have been proven partly attributing to its autophagy-inducing function (Nys et al., 2013), like SJ02’s protection of the endothelial cells against Crohn’s disease, a severe GI disorder. Besides, GHX05, another active ingredient, protects the gastric epithelial cells from the urease injury induced by *H. pylori*, and thus exhibited potent anti-bacterial activities in rats (Yu et al., 2015). It also has a wide spectrum of other biological activities, including anti-inflammation, oxidative balance regulation and the enhancement of gastric mucosa defense, etc. (Yu et al., 2015). In addition, GHX01, GHX06, GHX07, HP01, and HP02 as mentioned in previous section, all possess proper anti-bacterium activities. Thus, we speculate that these molecules that are associated with the activation of autophagy constitute the substance basis of HXZQ for its anti-bacterial function.

In addition, a few targets of HXZQ in Imm-C-T network are also observed getting involved in autophagy or anti-*H. pylori* processes. Actually, four of them, including GSK3B, NOS2, NOS3, have been proven capable of inducing the initiation of autophagy through inhibiting PI3K/Akt pathway (Heras-Sandoval et al., 2014). Videlicet, an active component of HXZQ may activate the autophagy process by targeting these proteins, and thus relieve the upset of GI system.

For instance, GSK3 is a highly conserved, constitutively active serine/threonine protein kinase. GSK3B, as a central regulator of inflammatory response, play roles in immune system against viral, fungal and parasitic infections (Wang et al., 2014). The pharmacological inhibition of GSK3B in TLR4-stimulated macrophages, may increase IFN-β production which has an important role in cell growth and differentiation. During the invasion process of *H. pylori*’s invading, the germ exploits GSK3B, seeking to avoid the immune system (Nakayama et al., 2009). Therefore, the regulation of GSK3B may protect host cells from *H. pylori*’s infection, making GSK3B a therapeutic target for the prevention of *H. pylori*-driven gastric disorder (Wang et al., 2014). Factually, GSK3B’s inactivation suppressed the *H. pylori*-induced pernicious biological activities. In Imm-C-T network, GSK3B interacts with 64 active ingredients of HXZQ, indicating its essential roles in eradication of *H. pylori*.

As is known to all, NO is closely related to human immune system that it induces or suppresses apoptosis as a toxic or immune regulatory media. Accompanied by NO’s production, the immune system is activated to fight against the invading bacteria. NO’s transmitting depends on its concentration or chemical reactivity rather than receptors. To initiate immune system, NO needs to be generated in great numbers to maintain high levels for sustained period of time. Therefore, its concentration and sustained time matter for normal immune response. NOS2 and NOS3, two forms of nitric oxide synthases, are in charge of NO productions, where NOS2 produces high concentrations of NO, which synthesis sustains for hours or days or even longer, whereas NOS3 only intermittently generates NO (Coleman, 2001). In the present Imm-C-T network, 84 and 25 molecules act on NOS2 and NOS3, respectively, indicating that HXZQ tends to produce sustainable and large concentration of NO to exert therapeutic effects on FD. Thus, regulating the production of NO to modulate the immune system through NOS2 and NOS3 targets should also account for the immune regulation function of HXZQ.

Actually, HXZQ formula is a very famous and classical prescription for the treatment of heat wet cold. Heat wet cold is a kind of exogenous cold disease caused by sudden wind, cold or dampness in summer, with main clinical symptoms of fever, dizziness, encephalalgia, tiredness, thirsty, chest tightness, nausea etc. Being a relatively mild disorder with a benign prognosis, heat wet cold belongs to categories of upper respiratory tract infection and influenza. The upper respiratory tract infection is induced by viral and bacterial infections that certain viruses, like rhinovirus, *adenovirus* (ADV), *influenza* virus, *coxsackie* virus (CVB3) and coronavirus, often lead to viral upper respiratory tract infection. In fact, the extract of *Jun* herb *GHX*, patchouli oil, has been proved possessing proper antiviral effects both *in vivo* and *in vitro*. For instance, it inhibits H1N1, CVB3 and ADV with concentrations of 0.088, 0.080, and 0.084 mg/ml, respectively (Wei et al., 2012). And the main active ingredient of *GHX*, patchouli alcohol (GHX05), not only has anti-Coxsackie virus, adenovirus, and *influenza* A virus capacity, but also shows higher potency than certain finished drugs. For example, ribavirin, a marketed medicine in prevention of virosis, inhibits H1N1, CVB3 and ADV with concentrations of 0.078, 0.067, and 0.063 mg/ml, respectively. Whereas, patchouli alcohol inhibits H1N1, CVB3, and ADV with even lower concentrations as 0.031, 0.063, and 0.063 mg/ml, respectively. And it has also been validated that patchouli alcohol exerts anti-adenovirus activity through interacting with Hexon, a target responsible for translating the capsid protein of adenovirus (Li et al., 2011). All these results demonstrate that anti-virus is an important mechanism of HXZQ for the treatment of heat wet cold.

In summary, the modulation function on immune system through the autophagy activation, the anti-bacteria (especially *H. pylori*) and antiviral processes, is also one mechanism accounting for HXZQ’s clinical protection efficacy for GI system.

##### The gastrointestinal motility regulation function

**FIGURE 10:**
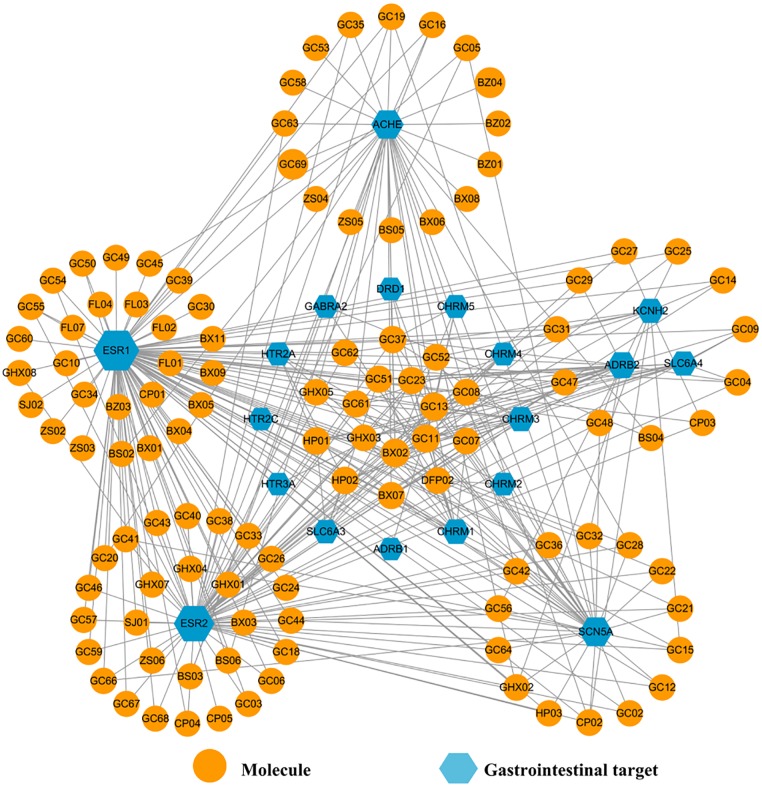
The Gas-C-T network of HXZQ formula, where hexagons and circles represent the GI motility-related targets and corresponding interacting compounds, respectively. The edges represent the mutual relations among the targets and chemicals. Node size is proportional to its degree.

Poor digestion, caused by insufficient gastroenteric movements, is a main symptom of FD, and hence it is necessary to promote the GI motility of the patients for treating this disease ultimately. Thus, presently a Gas-C-T network was constructed employing all 19 GI motility-related targets (represented by blue hexagons in Figure 10) and corresponding interacting compounds (orange circles) for exploring the function of HXZQ in promoting the GI motility in FD treatment.

In Gas-C-T network, the average degrees of these gastroenteric targets and components are 17 and 3, respectively, demonstrating complex interactions among multi-chemicals and multi-targets of HXZQ. Among them, three targets, ESR1, ESR2, and ACHE receptors, outstand due to their high connection degree indicating their pivotal roles in improving the gastrointestinal vitality, which has been validated by experimental facts actually. For instance, ESR1 and ESR2 are two subtypes of estrogen receptors who have also the highest degree as 100 and 75, respectively, in the net. In distribution, ER subtypes are detected not only in reproductive systems like mammary gland, uterus, ovary and prostate, but also in GI tissues such as fundus, antrum, and duodenum (Campbell-Thompson, 1997), indicating their potential roles in regulation of GI system. In function, they are in charge of mediating the action of various estrogens, which actually have been proven with inhibition effects on intestinal motility. Thus, ESR1 and ESR2 are involved in the mediation of colonic motility (Choijookhuu et al., 2016), where ESR2 is the predominant ER type in intestinal tract which inhibits A-type K^+^ currents of intestines smooth muscle cells and regulates the excitability of smooth muscle (Beckett et al., 2006). Similar to ESR2, ESR1 regulates the GI motility. As for ACHE, it is actually also a target of some western medicines for treating GI-related diseases (Serralheiro et al., 2013). For instance, two ACHE inhibitors, *neostigmine* and *metoclopramide*, are capable of reversing the impairment of gastrointestinal motility and treating gastric motility dysfunctions, respectively. Presently, ACHE interacts with 33 active molecules in Gas-C-T net. These all imply that ER receptors and ACHE may also account for the GI vitality regulation functions of HXZQ on FD treatment.

It is worthy of noting that some proteins, such as CHRM1 and DRD1, also exhibit favorable effects on GI disorders despite of their not so large degree. For instance, CHRM (M1 ∼ M5) are five distinct subtypes of muscarinic acetylcholine receptors, all of which have demonstrated as promising therapeutic targets for GI diseases (Matsui et al., 2002). Muscarinic receptors are widely expressed in smooth muscle in GI tract. The principle subtypes on the sarcolemma are CHRM2 and CHRM3. The activation of CHRM2 decreases the opening times of a potassium channel activated by β-adrenoceptor agonists, also attenuating the relaxation induced by the sympathetic systems (Eglen, 2001). In addition, in smooth muscle, CHRM3 receptor mediates the phosphoinositide hydrolysis and Ca^2+^ mobilization which contracts smooth muscle directly. Presently, it is discovered that HP01 and HP02, two out of the eleven components of HXZQ who act on CHRM3, are involved in the regulation of GI motility (as discussed previously). Therefore, we speculate that the other 9 molecules may also possess potentials in participating in the GI regulation. As to DRD1, one of the dopamine receptors, it is widespread in enteric nervous system like gastroesophageal junction, stomach, pylorus, small intestine and colon (Feng et al., 2013). DRD1’ substrate, namely, dopamine, reduces human gastric pressure and motility. DA antagonists like *domperidone* exhibit proper regulation functions on GI motility. Actually, it is through two actions, i.e., the contraction of the circular smooth muscle layer and/or the relaxation of the longitudinal smooth muscle layer (Vaughan et al., 2000), that DA receptors fulfill their direct modulations on the gastric smooth muscle cells responses. In this work, three chemicals (BX02, GC11, and GC13) act on DRD1 receptor. Although no experimental results have been reported, the connections among these molecules and DRD1 indicate the potential of these three compounds for treating GI motility disorders. And thus, the regulation of GI vitality by targets like CHRM (1 ∼ 5) and DRD1 may also be part of the mechanism of action of HXZQ for FD treatment.

It is well known that GI motility is regulated by three factors, i.e., the intact immune system, the enteric nerves, as well as the smooth muscle cells (Locke et al., 2006). Since the influence of immune system has been discussed in the analysis of Imm-C-T network, the impacts of two other factors, enteric nerves and smooth muscle cells, on GI motility are analyzed here.

For enteric nerves, some low-degreed targets produce positive impacts on FD treatment through acting on vagal afferents, which regulate the digestive system. Actually, vagal afferents are a kind of nerves that are extensively distributed in digestive tract from esophagus to colon. Since their function is to signal the initiation of several GI bio-processes including distension, contraction or relaxation of gastric smooth muscle, vagal afferents are often implicated in the flex control of the secretion and motility function of GI tract, and thus reflex FD (Andrews and Sanger, 2002). From Gas-C-T net, it is observed that vagal afferents affect several receptors by either enhancing (e.g., 5-HT3 receptor) or reducing (like κ-opioid and GABAB receptors) their activities, and in this way modulate the function of these proteins on GI motility regulation. These targets of vagal afferents, like 5-HT3 receptor, exert considerable influence on the regulation of GI motility. Over the past decade, 5-HT3 receptor antagonists (e.g., granisetron and ondansetron) have been used in the treatment of acute phase of emesis, post-operative nausea and vomiting. Presently, in Gas-C-T net, molecule maackiain (GC08) acts on HTR3A, indicating its potential capability of controlling GI motility through activating the vagus.

As to another type of target of vagal afferents, i.e., the κ-opioid or GABAB receptors, recorded researches have validated the importance of their activation for vagal afferent. GABAB is expressed on gastric vagal afferent neurones and reversibly inhibits gastric vagal mechanoreceptor responses to distension (Smid et al., 2001). There is a dense distribution of GABAB receptor along central vagal pathways in the nucleus tractus solitarii and dorsal vagal nucleus. GABAB receptor agonists, like baclofen, reduce the triggering of transient lower esophageal sphincter relaxations and thereby inhibit the gastroesophageal reflux in human body (Partosoedarso et al., 2001). Presently, three molecules including atractylenolide II (BS03), atractylenolide I (BS04) and arecolidine (DFP02) act on this target, indicating their potentials on the regulation of GI motility. In summary, the modulation of GI activity through regulating enteric nerves is an important factor for HXZQ contributing to the treatment of GI diseases.

Another factor that influences the GI motility is the smooth muscle cells, thus those proteins that regulate the function of these cells may also be potential targets for FD treatments. In Gas-C-T net, two ion channels that exist in smooth muscle cells, i.e., KCNH2 (encoded HERG potassium channel) and SCN5A (encoded Nav1.5 sodium channel) attract our attention. Actually, the importance of the first channel, namely, K^+^ channels, in regulating muscle tone and contractility of stomach has long been highlighted by recent studies that the activation or inhibition of K^+^ channels generates profound relaxations or inhibition of gastric smooth muscle (Locke et al., 2006). Specifically, in GI diseases, K^+^ channel activators facilitate the muscle relaxant activity. Thus, these channels ameliorate the accommodative function of proximal stomach. In irritable bowel syndrome with diarrhea, K^+^ channel activation is capable of reducing propulsive motor activity by relaxing both the circular muscle and the taenia coli (Currò, 2014). In this net, 11 active compounds interact with KCNH2. Among them, nobiletin (CP03) exerts suppressive effects on colon inflammation through down-regulation of cytokines as well as inflammation mediators, and decreases the intestinal epithelial permeability and restoration of barrier function (Xiong et al., 2015). Besides, CP03 bi-direction regulates jejuna contractility through the modulation of enteric nervous system, that is, it reflexes the high-contracting GI smooth muscle while exciting the low-contracting one (Yao et al., 2008). Although the biological efficacy of other 10 molecules have not been experimentally validated, we assume they may also possess potential bioactivities about regulating GI motility.

As to another ion channel, SCN5A, it is closely related to severe, frequent and chronic disorders like abdominal pain (Locke et al., 2006). SCN5A is expressed in circular smooth muscle cells and interstitial cells of Cajal. Factually, SCN5A impacts smooth muscle cells in both direct and indirect ways. The direct way is related with SCN5A’s inhibition, which hyperpolarizes human intestinal circular smooth muscle cells. Whereas, the indirect way is through an electrical slow wave which is generated by interstitial cells and tightly associated with the motility of GI tract. When SCN5A is inhibited, this slow wave’s rate of rise is slowed and its frequency is decreased, which finally ends up with the contraction of smooth muscles. Consequently, whether KCNH2 or SCN5A ion channels, they both are essential targets responsible for regulation of human intestinal motility (Ou et al., 2003). Presently, 36 active compounds act on SCN5A, similar to KCNH2, indicating that their potential therapeutic effects for FD treatment that they may modulate GI motility through acting on ion channels.

In summary, we find that it is through three mechanisms of action that HXZQ exerts therapeutic effects for FD treatment, i.e., the anti-inflammation, the immune protection (through autophagy activation or anti-bacteria actions like *H. pylori*) and the GI motility regulation (which is primarily dependent upon the regulation of enteric nerves and smooth muscle cells) based on network pharmacology analysis of the three C-T networks.

#### Target-Pathway Network

By mapping the targets to related pathways, we find that FD treatment is mostly related to four pathways in mechanism, i.e., PI3K-Akt, TLRs, JAK-STAT and Calcium signaling pathways. Therefore, pathway analysis is also conducted with purpose to deeply comprehend the mechanisms of HXZQ for FD treatment.

**FIGURE 11:**
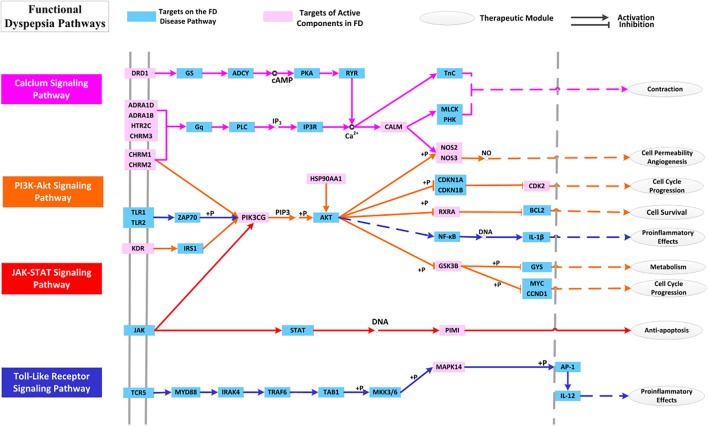
The distribution of target proteins on FD-related essential pathways. The line color corresponds to the pathway’s color.

PI3K, the key component of PI3K-Akt signaling pathway, is a lipid kinase abundant in leucocytes and regulates a wide variety of cellular processes including cellular growth, migration and proliferation. PI3K-Akt signaling pathway negatively modulates LPS-induced acute inflammatory responses. Factually, its inhibition enhances the activation of NF-κB, AP-1, and Egr-1 transcription factors as well as expression of TNF-α, IL-6 and tissue factor in human monocytic cells (Huang et al., 2011), which results in impaired immune responses and reduced susceptibility to autoimmune and inflammation (Andeol et al., 2018). This pathway is also involved in the regulation of autophagy in immune response, so as to remove bacteria from gastrointestinal infections. Thus, its regulation is crucial to the treatment of GI disorders. Presently, 9 targets are involved in PI3K-Akt pathway as important regulator of immune system, including CHRM1, CHRM2, KDR, PIK3CG, HSP90AA1, GSK3B, NOS3, RXRA, and CDK2 (Figure 11). Among them, the crucial roles of all proteins (except GSK3B) as anti-inflammation and immune regulation targets have already been discussed previously. While for GSK3B, a downstream target of Akt, it generates inflammatory cytokines and is involved in the immune system against invading pathogens, and in this way controls the cell survival and cell cycle progression (Laprise et al., 2004). Thus, all this indicates that PI3K-Akt pathway is a key channel for regulation of the anti-inflammation and immune protection functions of HXZQ formula.

As to JAK-STAT signaling pathway, it is partly the center of transduction of numerous signals for homeostasis and immune function, and is significant for a wide array of cytokines and growth factors (Coskun et al., 2013). JAK activation induces cell proliferation, differentiation, migration and apoptosis, and these cellular events are critical to immune development. Except for its roles in the regulation of key cellular activities, the JAK-STAT signaling pathway has also been implicated in the mechanism of inflammatory bowel diseases (Alegot et al., 2018). For each T cell subset, certain particular STAT is assigned. STAT protein can be activated by those cytokines presented in immune response which leads to a regulation on the balance of T cells and eventually contributes to the immune-protection and inflammation-alleviating of GI tracts. Presently, PIK3CG and PIMI participate in the regulation of JAK-STAT signaling pathway (Rawlings, 2004). PIM1 is a serine/threonine-protein kinase, which is in charge of the controls cell survival, proliferation, differentiation and death (Shin et al., 2012). PIM1 kinase, expressed in human eosinophils, also contributes to the survival of T cells in immune cells, and therefore participates in the regulation of immune system to treat various diseases. Thus, we speculate that PIM1 has potential GI protective effects against bacterial damage by regulating the JAK-STAT signaling pathway.

The third pathway involves TLRs signaling pathway, which are also important in modulation of both inflammatory and immune responses (Takeda, 2004). TLRs, expressed in immune cells, astrocytes and microglia, are crucial in early host defense against invading pathogens. TLRs recognize those microbial structures that have been saved in memory including the bacteric lipopolysaccharide and viral RNA, and hence, participate in the microbial recognition to the activation of specialized antigen-presenting cells in T lymphocyte (Akira and Takeda, 2004). Pathogen activates TLR signaling which then results in corresponding immune responses against the microbial infections. In enteroendocrine cells, TLR expression promotes the elimination of pathogens (Abreu, 2010) and thus TLR signaling pathway is important for inducing the immune response against the GI bacteria challenge, such as *H. pylori* and *coxsackie virus*. TLR signaling has also been implicated in epithelial cell proliferation, tight junctions’ maintenance and antimicrobial peptide expression, and TLR receptors are crucial for maintaining arobust enteric epithelial barrier. Presently, three targets, i.e., PIK3CG, MAPK14, and GSK3B, are involved in the regulation of TLRs signaling. And in HXZQ formula, GHX06 as an active ingredient acts on these three targets, which corresponds well to the experimental findings that GHX06 exerts its anti-oxidative and anti-inflammatory properties by inhibiting TLRs signaling (Ji et al., 2017).

This all indicates that the regulation of TLRs signaling pathway incorporating targets GSK3B, MAPK14, and PIK3CG to produce anti-inflammatory effects as well as to enhance the immunity of human body is also a reason for the curative effects of HXZQ on FD treatment.

Since neural factor is also crucial to FD treatment (Talley and Ford, 2015), calcium signaling pathway, the fourth signaling path, is also key for its close association with the treatment of GI motility insufficiency. This pathway regulates the synaptic transmission, and takes part in the neurosecretion and neuronal excitability. Besides, as a critical second messenger, Ca^2+^ stimulates the cell migration and proliferation and thus regulates a wide variety of functions of GI epithelial cells (Rao et al., 2001). In fact, for the damaged GI surface barrier caused by inflammatory bowel disease and injured/erosive mucosa induced by *H. pylori* infection, increased Ca^2+^ concentration is beneficial for epithelial cells’ healing (Rao et al., 2012). Presently, 10 targets (like 5-HT, CHRM family and NOS) with certain interacting active ingredients all function through regulating the Calcium signaling pathway for the treatment of GI disorders. For example, polyamines stimulates both the gastric mucosal restitution and duodenal mucosal erosions through Ca^2+^ signaling, and are essential for stimulation of cell migration. The active molecule BS03 (atractylenolide I) also stimulates intestinal epithelial cell migration and proliferation via polyamine-mediated Ca^2+^ signaling pathway (Song et al., 2017). In a word, Calcium signaling pathway is critical FD-related pathway in the regulation of GI motility.

Biological cross-talk refers to instances where one or more components of one signal transduction pathway affects another pathway (Kunkel and Brooks, 2002). Due to the existence of overlapping hubs, cross-talk often exists which links various pathways into an adaptable complex network. In Figure 11, multiple interactions are observed among these pathways through regulating one hinge protein, i.e., PIK3CG. As previously mentioned, PIK3CG serves as an important anti-inflammation target. Despite of its relatively low connection degree, it is still essential for FD treatment due to its pivotal position of cross-talk in the pathway network. Actually, these pathways are bonded together to regulate PIK3CG activities by mediating the intracellular signaling cascades. PIK3CG (PIKγ) is mainly expressed in leukocytes, and also presents at low concentration in smooth muscle cells. In function, PI3Kγ is necessary for chemokine-dependent migration of neutrophils, macrophages and mast cells to eliminate the infection (Marone et al., 2008). In HXZQ formula, PIK3CG is regulated by 14 active molecules such as GHX07 (Rutin), which is a gastro-protective natural flavonoid with anti-inflammatory activity and also used in the prevention of gastric mucosal ulceration.

In conclusion, PI3K-Akt signaling pathway participates in the process of inflammation and immune responses, whose regulation is helpful for the elimination of gastroenteritis. TLRs and JAK-STAT signaling pathways are mainly involved in the modulation of immune system, and their regulation may exert anti-inflammation activities and enhance immune ability to resist the bacteria infection. As to Calcium signaling pathway, it is mainly involved in learning or memory, as well as the contraction or relaxation of GI smooth muscle. In a word, it is just due to the complex pathway network composed by these pathways and their cross-talks that make HXZQ possess various and complementary functions for FD treatment.

### Computational Validation of Selected C-T Interactions

Since generally speaking, the inhibitory efficiency of a ligand is closely associated with the number and strength of its binding forces with its receptor (Wang et al., 2017), presently we explored the binding modes of several active ingredients with a GI regulating target CHRM3, an immunological target GSK3B and an anti-inflammatory target PTGS2 through molecular docking analysis as probes for validation of the C-T interactions of the formula.

**FIGURE 12:**
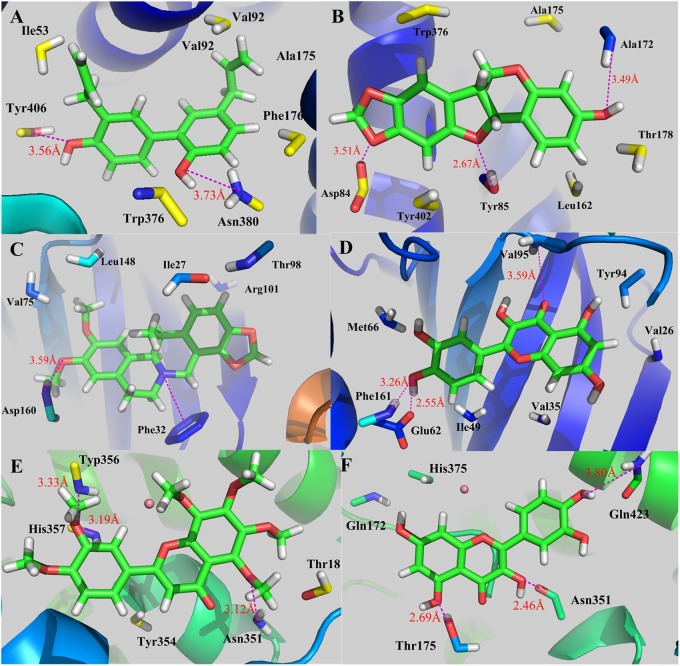
Binding explorations of selected C-T interactions. **(A)** CHRM3-HP02; **(B)** CHRM3-GC08; **(C)** GSK3B-BX02; **(D)** GSK3B-GHX06; **(E)** PTGS2-CP03; **(F)** PTGS2-GHX06. The molecules are displayed as ball and stick models, H-bonds are shown as dotted red lines with distance unit of Å. Atoms O and N are colored as red and blue, respectively.

Due to that HP02 has a unique array of pharmacological actions including the inhibition of multiple autonomic responses, firstly the molecular docking of HP02 with CHRM3 was performed, with results displayed in Figure 12A. From this figure, clearly two weak H-bonds are observed forming between the two –OH groups of HP02 and Tyr406 (3.56 Å) and Asn380 (3.73 Å), respectively. In addition, hydrophobic effects exist between the propyl side chain of HP02 and hydrophobic amino acid. All these interactions ensure the steady state of HP02 in the binding cavity, which well corresponds with the experimental result that HP02 potently inhibits M3 muscarinic receptors (CHRM3) (with EC_50_ = 5 μmol/l) and in this way regulates the GI motility (Wang et al., 2013). As for Figure 12B, GC08 (Maackiain) is fixed in the cavity through three H-bonds, including Asp84…O, Tyr85…O, and Ala172…O. Actually, GC08 is reported to exhibit great anti-GI bacteria action even at 10 μg/ml (Chan, 2002). The binding pattern explains why GC08 has such great biological activities.

As for BX02 (Cavidine), as the highest-degreed active compound, it participates in the regulation of human immune response as mentioned previously. As shown in Figure 12C, an H-bond between -OCH_3_ group and Asp160 (3.59 Å) and a π-cation interaction between the N atom of BX02 and the benzene of Phe32, are formed, respectively, in complex GSK3B-BX02. These interactions ensure the tight binding of BX02 and GSK3B which also accounts for BX02’s proper biological activities in immunomodulation. In Figure 12D, the H-bonding interactions between GHX06 and GSK3B, including Phe161…O, Glu62…O, and Val95…O, make GHX06-GSK3B complex remain a stable conformation. Actually, experiments have validated the interaction of GHX06 and GSK3B, whose strong binding forces further verify the proper antibacterial and antiviral effects as described in previous section (Bincy et al., 2016).

As shown in Figure 12E, CP03 is fixed the binding cavity of inflammatory target PTGS2 through three H-bonds with residues Typ356 (3.33 Å), His357 (3.19 Å) and Asn351 (3.12 Å). Actually, the binding of CP03 and PTGS2 explains CP03’s suppressing inflammation effects on GI system. As seen from Figure 12F, the complex PTGS2-GHX06 is stabilized by three H-bonds including the –OH groups with Gln423 (3.80 Å), Asn351 (2.46 Å), and Thr175 (2.69 Å), which further verifies GHX06’s anti-inflammation effects.

Based on this, the strong interactions between these active components and targets (CHRM3, GSK3B, and PTGS2) are the basis of these molecules’ biological activities.

## Discussion

Traditional Chinese Medicine is a precious heritage which has been practiced for three thousand years in Asia. Herbal medicine has been widely used for complicated disease treatment and is gradually becoming acceptable as alternative medicine worldwide. HXZQ is one of acclaimed TCM preparations in China which has been used in GI diseases treatment for thousands of years. The advantages of HXZQ for treating GI diseases mainly concentrate on the following three aspects. Firstly, corresponding to the present mechanism analysis results, HXZQ formula have multiple pharmacological functions including anti-inflammation, immune-protection and gastrointestinal motility regulation effects. Thus, HXZQ has been clinically applied in treating a few (instead of single) GI diseases which include, mostly, three ones, i.e., the gastroenteric cold, acute gastroenteritis and FD. Secondly, the active ingredients of HXZQ formula are diverse in both species and structures, and are large in number, which greatly reduce the possibility of drug resistance. Lastly, *GC* has antidote effects, which may account for the less side effects and low toxicity of HXZQ formula. And it is just due to the mutual promotion of the herbs ranking according to the compatibility principle of “*Jun-Chen-Zuo-Shi*,” HXZQ formula exert good polypharmacology activities. Presently, a systems pharmacology approach which contains ADME screening, targets prediction, network analysis and pathway screening was utilized, with purpose to probe the “multi-compound, multi-target, and multi-pathway” properties of HXZQ formula and the pathogenesis of FD from molecular to pathway levels. The main results are:

(1) 132 chemicals out of 1,192 components in 11 herbs, and 48 proteins/enzymes are identified as candidate compounds and FD-related targets of HXZQ prescription, respectively. And the systematic use of these active ingredients may supply useful clues on the combination therapies for FD treatment.

(2) Based on the traditional theory about the roles of the main and auxiliary herbs of TCM and the consideration about the distribution of active components in ingredient herbs and the contribution of their specific activities to the treatment of FD of HXZQ, this work interprets the combination rule of “*Jun-Chen-Zuo-Shi*” which HXZQ formula conforms to. That is, *GHX* possesses several beneficial bioactivities which direct impact the curative effects for FD treatment, and thus serves as *Jun* drug. *ZS* and *BZ* exert anti-bacterial effects and are also responsible for reliving major accompanying symptoms like stomach pain and gastro spasm of FD, hence are used as *Chen* drug. The treatment of minor symptoms, like abdominal distension, belching, nausea and vomiting, is undertaken by *BX*, *HP*, *FL*, *SJ*, *CP*, *BS*, and *DFP*, as *Zuo* drugs. Whereas, *GC* is employed as *Shi* drug because of not only its detoxication capacity, but also its ability in influencing the ADME properties of other herbs, as well as the relationship among other herbs.

(3) The mechanism of action of HXZQ formula for treating FD is fulfilled through its three function modules, i.e., the suppression of inflammation, the intensification of immunological response and the regulation of GI motility. And the implementation of these functions rely on smooth run of the complex bio-pathway network which includes especially four pathways, namely, PIK-AKT signaling pathway, JAK-STAT signaling pathway, Toll-like signaling pathway and Calcium signaling pathway.

In summary, GI disorders are multi-factors caused complex diseases. For TCMs, such as HXZQ, due to their multi-components and multi-targets characteristics, they have advantages especially in the multimodality treatment of GI diseases from molecule-tissue-organ-body multi-levels as a holistic medicine. This study will not only facilitate the development of phytomedicines in modern medicine, but also provide a modern interpretation of traditional TCM theory of “*Jun-Chen-Zuo-Shi*”.

Most Chinese herbal medicines come from plants, which often possess dozens or even hundreds of various chemical components with diversified structures. And this makes, factually, the determination of TCM content an indispensable while quite challenging work in TCM research. At present, although series of analysis methods, such as high performance liquid chromatography (HPLC), gas chromatography, thin-layer chromatography, molecular absorption spectrophotometry (ultraviolet), titration, etc., have been developed and applied on determination of TCM ingredients’ contents, they usually aim at merely one or several TCM components. Even so, these methods themselves also exist certain limits which makes the determination of all TCM components almost impossible (Zhong et al., 2015). As a matter of fact, the development of testing methods for TCM content is itself a hot topic in Chinese medicine research. Thus, due to the lacking of the specific content data of the full components of herbs, this work did not carry out a deep mathematical analysis on the composition content. Whereas, the influence of the contents has also been considered for that those abundant ingredients are also assumed as candidate compounds even if with relatively low oral availability or other ADME properties. Thus, in the near future, with more integrated and full data of TCM’s ingredient content becoming available, the investigation on influence of TCM content on its mechanism of action should also be a significant job.

## Author Contributions

MZ conducted the statistical analysis and wrote the paper. YC implemented the methods and conducted the analysis. SC, SZ, and YL contributed to the paper and provided guidance. WX provided new ideas in statistical analysis section. All authors read and approved the final manuscript.

## Conflict of Interest Statement

The authors declare that the research was conducted in the absence of any commercial or financial relationships that could be construed as a potential conflict of interest. The handling Editor and reviewer, S-BS, declared their involvement as co-editors in the Research Topic, and confirm the absence of any other collaboration. In addition, two reviewers, S-BS and XC, respectively declared shared affiliations, but no collaboration, with two of the authors, LY and YC, to the handling Editor.

## Footnotes

1 http://lsp.nwu.edu.cn/articles.php?id=2

2 http://www.organchem.csdb.cn

3 http://tcm.cmu.edu.tw/

4 https://omictools.com/tcmid-tool

5 http://www.ncbi.nlm.nih.gov/pccompound

6 http://www.drugbank.ca/

7 http://bidd.nus.edu.sg/group/ttd/

8 http://www.pharmgkb.org

9 http://www.uniprot.org/

10 https://david.ncifcrf.gov/

11 http://www.genome.jp/kegg/

12 http://www.rcsb.org/

## Abbreviations

DL: drug-likeness
FD: functional dyspepsia
GDs: gastrointestinal diseases
GI: gastrointestine
GO: Gene Ontology
*H. pylori*: *Helicobacter Pylori;* HXZQ *Huo-xiang-zheng-qi;* IL-1β interleukin-1 beta
IL-6: interleukin-6
NSAIDs: non-steroidal anti-inflammatory drugs
OB: oral bioavailability
PGE_2_: prostaglandin E_2_
*P*-gp: *p*-glycoprotein
RF: random forest
SEE: standard error of estimate
SVM: supporting vector machine
TCM: Traditional Chinese medicine
TLRs: Toll-like receptors
TNF-α: tumor necrosis factor α

## References

[B1] Abreu M. T. (2010). Toll-like receptor signalling in the intestinal epithelium: how bacterial recognition shapes intestinal function. *Nat. Rev. Immunol.* 10 131–144. 10.1038/nri2707 20098461

[B2] Akira S. Takeda K. (2004). Toll-like receptor signalling. *Nat. Rev. Immunol.* 4 499–511. 10.1038/nri1391 15229469

[B3] Alegot H. Pouchin P. Bardot O. Mirouse V. (2018). Jak-Stat pathway induces *Drosophila* follicle elongation by a gradient of apical contractility. *Elife* 7:e32943. 10.7554/eLife.32943 29420170 PMC5805408

[B4] Andeol Y. Bonneau J. M. Gagne L. Jacquet K. Rivest V. Huot M. (2018). The phosphoinositide 3-kinase (PI3K) pathway and glycogen synthase kinase-3 (GSK-3) positively regulate the activity of metal-responsive transcription factor-1 (MTF-1) in response to zinc ions. *Biochem. Cell Biol.* 10.1139/bcb-2018-0073 [Epub ahead of print]. 29707960

[B5] Andrews P. L. Sanger G. J. (2002). Abdominal vagal afferent neurones: an important target for the treatment of gastrointestinal dysfunction. *Curr. Opin. Pharmacol.* 2 650–656. 10.1016/S1471-4892(02)00227-8 12482726

[B6] Bae E. A. Han M. J. Kim D. H. (1999). In vitro anti-*Helicobacter pylori* activity of some flavonoids and their metabolites. *Planta Med.* 65 442–443. 10.1055/s-2006-960805 10454900

[B7] Beckett E. A. H. McCloskey C. O’Kane N. Sanders K. M. Don Koh S. (2006). Effects of female steroid hormones on A-type K + currents in murine colon. *J. Physiol.* 573 453–468. 10.1113/jphysiol.2006.107375 16581861 PMC1779718

[B8] Bincy B. Priya A. Walaa A. Al Homedi Z. Vijayan R. (2016). Structural insights into the polypharmacological activity of quercetin on serine/threonine kinases. *Drug Des. Dev. Ther.* 10 3109–3123. 10.2147/DDDT.S118423 27729770 PMC5045902

[B9] Buzas G. M. (2007). Functional dyspepsia: the past, the present and the Rome III classification. *Orv. Hetil.* 148 1573–1579. 10.1556/OH.2007.28131 17686677

[B10] Campbell-Thompson M. L. (1997). Estrogen Receptor α and β Expression in Upper Gastrointestinal Tract with Regulation of Trefoil Factor Family 2 mRNA Levels in Ovariectomized Rats. *Biochem. Biophys. Res. Commun.* 2 478–483. 10.1006/bbrc.1997.7683 9388504

[B11] Chan M. (2002). Antimicrobial effect of resveratrol on dermatophytes and bacterial pathogens of the skin. *Biochem. Pharmacol.* 63 99–104. 10.1016/S0006-2952(01)00886-3 11841782

[B12] Chen Y. C. Shen S. C. Chen L. G. Lee T. J. Yang L. L. (2001). Wogonin, baicalin, and baicalein inhibition of inducible nitric oxide synthase and cyclooxygenase-2 gene expressions induced by nitric oxide synthase inhibitors and lipopolysaccharide. *Biochem. Pharmacol.* 61 1417–1427. 10.1016/S0006-2952(01)00594-9 11331078

[B13] Chinetti G. Fruchart J. C. Staels B. (2000). Review Peroxisome proliferator-activated receptors (PPARs): nuclear recep- tors at the crossroads between lipid metabolism and inflammation. *Inflamm. Res.* 49 497–505. 10.1007/s000110050622 11089900

[B14] Choijookhuu N. Hino S. Oo P. S. Batmunkh B. Hishikawa Y. (2016). The role of estrogen receptors in intestinal homeostasis and disease. *Recept. Clin. Investig.* 3 1–8. 10.14800/rci.1109

[B15] Chulada P. C. Thompson M. B. Mahler J. F. Doyle C. M. Gaul B. W. Lee C. (2000). Genetic disruption of Ptgs-1, as well as of Ptgs-2, reduces intestinal tumorigenesis in Min mice. *Cancer Res.* 60 4705–4708. 10987272

[B16] Chung D. Keles S. (2010). Sparse partial least squares classification for high dimensional data. *Stat. Appl. Genet. Mol.* 9:17. 10.2202/1544-6115.1492 20361856 PMC2861314

[B17] Coleman J. W. (2001). Nitric oxide in immunity and inflammation. *Int. Immunopharmacol.* 1 1397–1406. 10.1016/S1567-5769(01)00086-8 11515807

[B18] Coskun M. Salem M. Pedersen J. Nielsen O. H. (2013). Involvement of JAK/STAT signaling in the pathogenesis of inflammatory bowel disease. *Pharmacol. Res.* 76 1–8. 10.1016/j.phrs.2013.06.007 23827161

[B19] Currò D. (2014). K + channels as potential targets for the treatment of gastrointestinal motor disorders. *Eur. J. Pharmacol.* 733 97–101. 10.1016/j.ejphar.2014.03.049 24726846

[B20] Deretic V. Saitoh T. Akira S. (2013). Autophagy in infection, inflammation and immunity. *Nat. Rev. Immunol.* 13 722–737. 10.1038/nri3532 24064518 PMC5340150

[B21] Eglen R. M. (2001). Muscarinic receptors and gastrointestinal tract smooth muscle function. *Life Sci.* 68 2573–2578. 10.1016/S0024-3205(01)01054-2 11392628

[B22] Feng X. Li Y. Li L. Li X. Zheng L. Zhang X. (2013). Dopamine D1 receptors mediate dopamine-induced duodenal epithelial ion transport in rats. *Transl. Res.* 161 486–494. 10.1016/j.trsl.2012.12.002 23276732

[B23] Fu X. Chou J. Li T. Zhu P. Li J. Yin C. (2018). The JAK2/STAT3 pathway is involved in the anti-melanoma effects of atractylenolide I. *Exp. Dermatol.* 27 201–204. 10.1111/exd.13454 29078004

[B24] Fukai T. Marumo A. Kaitou K. Kanda T. Terada S. Nomura T. (2002). Anti-*Helicobacter pylori* flavonoids from licorice extract. *Life Sci.* 71 1449–1463. 10.1016/S0024-3205(02)01864-7 12127165

[B25] Gao Y. Liu F. Fang L. Cai R. Zong C. Qi Y. (2014). Genkwanin inhibits proinflammatory mediators mainly through the regulation of miR-101/MKP-1/MAPK pathway in LPS-activated macrophages. *PLoS One* 9:e96741. 10.1371/journal.pone.0096741 24800851 PMC4011752

[B26] Grobelny B. T. London D. Hill T. C. North E. Dugan P. Doyle W. K. (2018). Betweenness centrality of intracranial electroencephalography networks and surgical epilepsy outcome. *Clin. Neuropysiol.* 129 1804–1812. 10.1016/j.clinph.2018.02.135 29981955

[B27] Gu L. Wu T. Wang Z. (2009). TLC bioautography-guided isolation of antioxidants from fruit of Perilla frutescens var. acuta. *LWT Food Sci. Technol.* 42 131–136. 10.1016/j.lwt.2008.04.006

[B28] Harnish D. C. Albert L. M. Leathurby Y. Eckert A. M. Ciarletta A. Kasaian M. (2004). Beneficial effects of estrogen treatment in the HLA-B27 transgenic rat model of inflammatory bowel disease. *Am. J. Physiol. Gastrointest. Liver Physiol.* 286 G118–G125. 10.1152/ajpgi.00024.2003 12958017

[B29] He Y. H. Zhao H. Y. Liu Z. L. Lu C. Luo X. J. Lin S. Q. (2006). Effects of huoxiangzhengqi liquid on enteric mucosal immune responses in mice with Bacillus dysenteriae and *Salmonella typhimurium* induced diarrhea. *World J. Gastroenterol.* 12 7346–7349. 10.4314/tjpr.v12i4.18 17143954 PMC4087496

[B30] Heras-Sandoval D. Pérez-Rojas J. M. Hernández-Damián J. Pedraza-Chaverri J. (2014). The role of PI3K/AKT/mTOR pathway in the modulation of autophagy and the clearance of protein aggregates in neurodegeneration. *Cell. Signal.* 26 2694–2701. 10.1016/j.cellsig.2014.08.019 25173700

[B31] Hichri I. Barrieu F. Bogs J. Kappel C. Delrot S. Lauvergeat V. (2011). Recent advances in the transcriptional regulation of the flavonoid biosynthetic pathway. *J. Exp. Bot.* 62 2465–2483. 10.1093/jxb/erq442 21278228

[B32] Hou Y. Lin S. Chao P. L. (2012). Liquorice reduced cyclosporine bioavailability by activating P-glycoprotein and CYP 3A. *Food Chem.* 135 2307–2312. 10.1016/j.foodchem.2012.07.061 22980806

[B33] Huang C. Y. Hsiao J. K. Lu Y. Z. Lee T. C. Yu L. C. (2011). Anti-apoptotic PI3K/Akt signaling by sodium/glucose transporter 1 reduces epithelial barrier damage and bacterial translocation in intestinal ischemia. *Lab. Invest.* 91 294–309. 10.1038/labinvest.2010.177 20975661

[B34] Ji C. Xu Y. Han F. Sun D. Zhang H. Li X. (2017). Quercetin alleviates thermal and cold hyperalgesia in a rat neuropathic pain model by inhibiting Toll-like receptor signaling. *Biomed. Pharmacother.* 94 652–658. 10.1016/j.biopha.2017.07.145 28787700

[B35] Juergens U. R. Dethlefsen U. Steinkamp G. Gillissen A. Repges R. Vetter H. (2003). Anti-inflammatory activity of 1.8-cineol (eucalyptol) in bronchial asthma: a double-blind placebo-controlled trial. *Respir. Med.* 97 250–256. 10.1053/rmed.2003.1432 12645832

[B36] Juergens U. R. Stöber M. Vetter H. (1998). Inhibition of cytokine production and arachidonic acid metabolism by eucalyptol (1.8-cineole) in human blood monocytes in vitro. *Eur. J. Med. Res.* 3 508–510. 9810029

[B37] Kang K. A. Zhang R. Piao M. J. Ko D. O. Wang Z. H. Kim B. J. (2008). Protective effect of irisolidone, a metabolite of kakkalide, against hydrogen peroxide induced cell damage via antioxidant effect. *Bioorg. Med. Chem.* 16 1133–1141. 10.1016/j.bmc.2007.10.085 17996449

[B38] Kim J. M. Yun-Choi H. S. (2008). Anti-platelet effects of flavonoids and flavonoid-glycosides from *Sophora japonica*. *Arch. Pharm. Res.* 31 886–890. 10.1007/s12272-001-1242-1 18704331

[B39] Knekt P. Kumpulainen J. Jarvinen R. Rissanen H. Heliovaara M. Reunanen A. (2002). Flavonoid intake and risk of chronic diseases. *Am. J. Clin. Nutr.* 76 560–568. 10.1093/ajcn/76.3.560 12198000

[B40] Kunkel B. N. Brooks D. M. (2002). Cross talk between signaling pathways in pathogen defense. *Curr. Opin. Plant Biol.* 5 325–331. 10.1093/ajcn/76.3.560 12179966

[B41] Kusters J. G. van Vliet A. H. M. Kuipers E. J. (2006). Pathogenesis of *Helicobacter pylori* Infection. *Clin. Microbiol. Rev.* 19 449–490. 10.1128/CMR.00054-05 16847081 PMC1539101

[B42] Langenbach R. Morham S. G. Tiano H. F. Loftin C. D. Ghanayem B. I. Chulada P. C. (1995). Prostagl and in-synthase-1 gene disruption in mice reduces arachidonic acid-induced inflammation and indomethacin-induced gastric-ulceration. *Cell* 83 483–492. 10.1016/0092-8674(95)90126-4 8521478

[B43] Laprise P. Langlois M. E. Boucher M. E. Jobin C. Rivard N. (2004). Down-regulation of MEK/ERK signaling by E-cadherin-dependent PI3K/Akt pathway in differentiating intestinal epithelial cells. *J. Cell. Physiol.* 199 32–39. 10.1002/jcp.10432 14978732

[B44] Lee J. Lee J. Y. Park J. H. Jung H. S. Kim J. S. Kang S. S. (2007). Immunoregulatory activity by daucosterol, a β-sitosterol glycoside, induces protective Th1 immune response against disseminated Candidiasis in mice. *Vaccine* 25 3834–3840. 10.1016/j.vaccine.2007.01.108 17335944

[B45] Lee J. H. Lee S. H. Kim Y. S. Jeong C. S. (2009). Protective effects of neohesperidin and poncirin isolated from the fruits of *Poncirus trifoliata* on potential gastric disease. *Phytother. Res.* 23 1748–1753. 10.1002/ptr.2840 19367677

[B46] Li S. Zhang B. (2013). Traditional Chinese medicine network pharmacology: theory, methodology and application. *Chin. J. Nat. Med.* 11 110–120. 10.3724/SP.J.1009.2013.00110 23787177

[B47] Li X. Xu X. Wang J. Yu H. Wang X. Yang H. (2012). A system-level investigation into the mechanisms of Chinese Traditional Medicine: compound Danshen Formula for cardiovascular disease treatment. *PLoS One* 7:e43918. 10.1371/journal.pone.0043918 22962593 PMC3433480

[B48] Li Y. Han C. Wang J. Xiao W. Wang Z. Zhang J. (2014). Investigation into the mechanism of *Eucommia ulmoides* Oliv. based on a systems pharmacology approach. *J. Ethnopharmacol.* 151 452–460. 10.1016/j.jep.2013.10.067 24239601

[B49] Li Y. Peng S. Chen H. Zhang F. Xu P. Xie J. (2012). Oral administration of patchouli alcohol isolated from Pogostemonis Herba augments protection against influenza viral infection in mice. *Int. Immunopharmacol.* 12 294–301. 10.1016/j.intimp.2011.12.007 22193241

[B50] Li Y. Wang J. Lin F. Yang Y. Chen S. S. (2017). A methodology for cancer therapeutics by systems pharmacology-based analysis: a case study on breast cancer-related traditional Chinese medicines. *PLoS One* 12:e0169363. 10.1371/journal.pone.0169363 28068355 PMC5222515

[B51] Li Y. Wang J. Xiao Y. Wang Y. Chen S. Yang Y. (2015a). A systems pharmacology approach to investigate the mechanisms of action of Semen Strychni and *Tripterygium wilfordii* Hook F for treatment of rheumatoid arthritis. *J. Ethnopharmacol.* 175 301–314. 10.1016/j.jep.2015.09.016 26386382

[B52] Li Y. Xian Y. Ip S. P. Su Z. R. Su J. Y. He J. J. (2011). Anti-inflammatory activity of patchouli alcohol isolated from Pogostemonis Herba in animal models. *Fitoterapia* 82 1295–1301. 10.1016/j.fitote.2011.09.003 21958968

[B53] Li Y. Zhang J. Zhang L. Chen X. Pan Y. Chen S. (2015b). Systems pharmacology to decipher the combinational anti-migraine effects of Tianshu formula. *J. Ethnopharmacol.* 174 45–56. 10.1016/j.jep.2015.07.043 26231449

[B54] Liao J. B. Wu D. W. Peng S. Z. Xie J. H. Li Y. C. Su J. Y. (2013). Immunomodulatory potential of patchouli alcohol isolated from *Pogostemon cablin* (Blanco) Benth (Lamiaceae) in Mice. *Trop. J. Pharm. Res.* 12 559–565. 10.4314/tjpr.v12i4.18

[B55] Lipinski C. A. Lombardo F. Dominy B. W. Feeney P. J. (2001). Experimental and computational approaches to estimate solubility and permeability in drug discovery and development settings. *Adv. Drug Deliv. Rev.* 46 3–26. 10.1016/S0169-409X(00)00129-0 11259830

[B56] Liu H. Wang J. Zhou W. Wang Y. Yang L. (2013). Systems approaches and polypharmacology for drug discovery from herbal medicines: an example using licorice. *J. Ethnopharmacol.* 146 773–793. 10.1016/j.jep.2013.02.004 23415946

[B57] Locke G. R. Ackerman M. J. Zinsmeister A. R. Thapa P. Farrugia G. (2006). Gastrointestinal symptoms in families of patients with an SCN5A-encoded cardiac channelopathy: evidence of an intestinal channelopathy. *Am. J. Gastroenterol.* 101 1299–1304. 10.1111/j.1572-0241.2006.00507 16771953

[B58] Loutrari H. (2004). Perillyl alcohol is an angiogenesis inhibitor. *J. Pharmacol. Exp. Ther.* 311 568–575. 10.1124/jpet.104.070516 15210838

[B59] Marone R. Cmiljanovic V. Giese B. Wymann M. P. (2008). Targeting phosphoinositide 3-kinase-Moving towards therapy. *Biochim. Biophys. Acta* 1784 159–185. 10.1016/j.bbapap.2007.10.003 17997386

[B60] Matsui M. Motomura D. Fujiwara T. Jiang J. Takahashi S. Manabe T. (2002). Mice lacking M-2 and M-3 muscarinic acetylcholine receptors are devoid of cholinergic smooth muscle contractions but still viable. 22, 10627-10632. *J. Neurosci.* 22 10627–10632. 10.1523/JNEUROSCI.22-24-10627.2002 12486155 PMC6758427

[B61] Mizushima N. (2007). Autophagy: process and function. *Gene. Dev.* 21 2861–2873. 10.1101/gad.1599207 18006683

[B62] Nakayama M. Hisatsune J. Yamasaki E. Isomoto H. Kurazono H. Hatakeyama M. (2009). *Helicobacter pylori* VacA-induced inhibition of GSK3 through the PI3K/Akt signaling pathway. *J. Biol. Chem.* 284 1612–1619. 10.1074/jbc.M806981200 18996844 PMC2615499

[B63] Niu X. Zhang H. Li W. Mu Q. Yao H. Wang Y. (2015). Anti-inflammatory effects of cavidine *in vitro* and *in vivo*, a selective COX-2 inhibitor in LPS-induced peritoneal macrophages of mouse. *Inflammation* 38 923–933. 10.1007/s10753-014-0054-4 25373916

[B64] Nys K. Agostinis P. Vermeire S. (2013). Autophagy: a new target or an old strategy for the treatment of Crohn’s disease? *Nat. Rev. Gastroenterol. Hepatol.* 10 395–401. 10.1038/nrgastro.2013.66 23591407

[B65] Ou Y. Strege P. Miller S. M. Makielski J. Ackerman M. Gibbons S. J. (2003). Syntrophin γ2 regulates SCN5A gating by a PDZ domain-mediated interaction. *J. Biol. Chem.* 278 1915–1923. 10.1074/jbc.M209938200 12429735

[B66] Parente L. Perretti M. (2003). Advances in the pathophysiology of constitutive and inducible cyclooxygenases: two enzymes in the spotlight. *Biochem. Pharmacol.* 65 153–159. 10.1016/S0006-2952(02)01422-3 12504791

[B67] Park J. Lee J. Jung E. Park Y. Kim K. Park B. (2004). In vitro antibacterial and anti-inflammatory effects of honokiol and magnolol against *Propionibacterium* sp. *Eur. J. Pharmacol.* 496 189–195. 10.1016/j.ejphar.2004.05.047 15288590

[B68] Park J. S. Woo M. S. Kim D. H. Hyun J. W. Kim W. K. Lee J. C. (2006). Anti-inflammatory mechanisms of isoflavone metabolites in lipopolysaccharide-stimulated microglial cells. *J. Pharmacol. Exp. Ther.* 320 1237–1245. 10.1124/jpet.106.114322 17194798

[B69] Partosoedarso E. R. Young R. L. Blackshaw L. A. (2001). GABA(B) receptors on vagal afferent pathways: peripheral and central inhibition. *Am. J. Physiol. Gastrointest. Liver Physiol.* 280 G658–G668. 10.1152/ajpgi.2001.280.4.G658 11254492

[B70] Peery A. F. Crockett S. D. Barritt A. S. Dellon E. S. Eluri S. Gangarosa L. M. (2015). Burden of gastrointestinal, liver, and pancreatic diseases in the United States. *Gastroenterology* 149 1731–1741.e3. 10.1053/j.gastro.2015.08.045 26327134 PMC4663148

[B71] Pei T. Zheng C. Huang C. Chen X. Guo Z. Fu Y. (2016). Systematic understanding the mechanisms of vitiligo pathogenesis and its treatment by Qubaibabuqi formula. *J. Ethnopharmacol.* 190 272–287. 10.1016/j.jep.2016.06.001 27265513

[B72] Rao J. N. Li L. Golovina V. A. Platoshyn O. Strauch E. D. Yuan J. X. (2001). Ca2 + -RhoA signaling pathway required for polyamine-dependent intestinal epithelial cell migration. *Am. J. Physiol. Cell Physiol.* 280 C993–C1007. 10.1152/ajpcell.2001.280.4.C993 11245616

[B73] Rao J. N. Rathor N. Zhuang R. Zou T. Liu L. Xiao L. (2012). Polyamines regulate intestinal epithelial restitution through TRPC1-mediated Ca2 + signaling by differentially modulating STIM1 and STIM2. *Am. J. Physiol. Cell Physiol.* 303 C308–C317. 10.1152/ajpcell.00120.2012 22592407 PMC3423028

[B74] Rawlings J. S. (2004). The JAK/STAT signaling pathway. *J. Cell Sci.* 117 1281–1283. 10.1242/jcs.00963 15020666

[B75] Rubis B. Paszel A. Kaczmarek M. Rudzinska M. Jelen H. Rybczynska M. (2008). Beneficial or harmful influence of phytosterols on human cells? *Br. J. Nutr.* 100 1183–1191. 10.1017/S0007114508981423 18445305

[B76] Saeidnia S. Manayi A. Gohari A. R. Abdollahi M. (2014). The story of beta-sitosterol- a review. *Eur. J. Med. Plants* 5 590–609. 10.9734/EJMP/2014/7764

[B77] Sekiya K. Okkuda H. (1982). Selective inhibition of platelet lipoxygenase by baicalein. *Biochem. Bioph. Res. Commun.* 3 1090–1095. 10.1016/0006-291X(82)91081-6 6807310

[B78] Serralheiro M. L. M. Falé P. L. Ferreira C. Rodrigues A. M. Cleto P. Madeira P. J. A. (2013). Antioxidant and anti-acetylcholinesterase activity of commercially available medicinal infusions after in vitro gastrointestinal digestion. *J. Med. Plants Res.* 7 1370–1378. 10.5897/JMPR13.4438

[B79] Shin Y. S. Takeda K. Shiraishi Y. Jia Y. Wang M. Jackson L. (2012). Inhibition of Pim1 Kinase Activation Attenuates Allergen-Induced Airway Hyperresponsiveness and Inflammation. *Am. J. Respir. Cell Mol.* 46 488–497. 10.1165/rcmb.2011-0190OC 22074702 PMC3359953

[B80] Singh M. Ganesha Rao R. S. (2009). Influence of sources and doses of N and K on herbage, oil yield and nutrient uptake of patchouli *Pogostemon cablin* (Blanco) Benth. in semiarid tropics. *Ind. Crop. Prod.* 29 229–234. 10.1016/j.indcrop.2008.05.005

[B81] Smid S. D. Young R. L. Cooper N. J. Blackshaw L. A. (2001). GABA(B)R expressed on vagal afferent neurones inhibit gastric mechanosensitivity in ferret proximal stomach. *Am. J. Physiol. Gastrointest. Liver Physiol.* 281 G1494–G1501. 10.1152/ajpgi.2001.281.6.G1494 11705755

[B82] Song H. Hou X. Li R. Yu R. Li X. Zhou S. (2017). Atractylenolide I stimulates intestinal epithelial repair through polyamine-mediated Ca 2 + signaling pathway. *Phytomedicine* 28 27–35. 10.1016/j.phymed.2017.03.001 28478810

[B83] Takeda K. (2004). Toll-like receptors in innate immunity. *Int. Immunol.* 17 1–14. 10.1093/intimm/dxh186 15585605

[B84] Talley N. J. Ford A. C. (2015). Functional Dyspepsia. *New Engl. J. Med.* 19 1853–1863. 10.1056/NEJMra1501505 26535514

[B85] Vaughan C. J. Aherne A. M. Lane E. Power O. Carey R. M. O’Connell D. P. (2000). Identification and regional distribution of the dopamine D1A receptor in the gastrointestinal tract. *Am. J. Physiol. Regul. Integr. Comp. Physiol.* 279 R599–R609. 10.1152/ajpregu.2000.279.2.R599 10938251

[B86] Wang A. Xiao Z. Zhou L. Zhang J. Li X. He Q. (2016). The protective effect of atractylenolide I on systemic inflammation in the mouse model of sepsis created by cecal ligation and puncture. *Pharm. Biol.* 54 146–150. 10.3109/13880209.2015.1024330 25853971

[B87] Wang H. Brown J. Martin M. (2011). Glycogen synthase kinase 3: a point of convergence for the host inflammatory response. *Cytokine* 53 130–140. 10.1016/j.cyto.2010.10.009 21095632 PMC3021641

[B88] Wang H. Kumar A. Lamont R. J. Scott D. A. (2014). GSK3β and the control of infectious bacterial diseases. *Trends Microbiol.* 22 208–217. 10.1016/j.tim.2014.01.009 24618402 PMC4003878

[B89] Wang H. J. Martin A. G. Chao P. K. Reichard R. A. Martin A. L. Huang Y. W. (2013). Honokiol blocks store operated calcium entry in CHO cells expressing the M3 muscarinic receptor: honokiol and muscarinic signaling. *J. Biomed. Sci.* 20:11. 10.1186/1423-0127-20-11 23432810 PMC3599152

[B90] Wang J. Li Y. Yang Y. Chen X. Du J. Zheng Q. (2017). A new strategy for deleting animal drugs from traditional chinese medicines based on modified yimusake formula. *Sci. Rep.* 7:1504. 10.1038/s41598-017-01613-7 28473709 PMC5431437

[B91] Wang Y. Zheng C. Huang C. Li Y. Chen X. Wu Z. (2015). Systems pharmacology dissecting holistic medicine for treatment of complex diseases: an example using cardiocerebrovascular diseases treated by TCM. *Evid. Based Complement. Alternat. Med.* 2015:980190. 10.1155/2015/980190 26101539 PMC4460250

[B92] Wei X. Peng C. Wan F. (2012). Study on the effect of anti-respiratory virus of Patchouli Oil in vitro. *Pharm. Clin. Chin. Herb. Med.* 28:6.

[B93] Williams C. S. Mann M. DuBois R. N. (1999). The role of cyclooxygenases in inflammation, cancer, and development. *Oncogene* 18 7908–7916. 10.1038/sj.onc.1203286 10630643

[B94] Wishart D. S. (2006). DrugBank: a comprehensive resource for in silico drug discovery and exploration. *Nucleic Acids Res.* 34 D668–D672. 10.1093/nar/gkj067 16381955 PMC1347430

[B95] Wu L. Wang Y. Li Z. Zhang B. Cheng Y. Fan X. (2014). Identifying roles of “Jun-Chen-Zuo-Shi” component herbs of QiShenYiQi formula in treating acute myocardial ischemia by network pharmacology. *Chin. Med.* 9:24. 10.1186/1749-8546-9-24 25342960 PMC4196468

[B96] Xiao Y. Liu Y. Yu K. Ouyang M. Luo R. Zhao X. (2012). Chinese Herbal Medicine Liu Jun Zi Tang and Xiang Sha Liu Jun Zi Tang for Functional Dyspepsia: Meta-Analysis of Randomized Controlled Trials. *Evid. Based Complement. Alternat. Med.* 2012:936459. 10.1155/2012/936459 23304226 PMC3530827

[B97] Xie D. P. Chen L. B. Liu C. Y. Zhang C. L. Liu K. J. Wang P. S. (2004). Arecoline excites the colonic smooth muscle motility via M-3 receptor in rabbits. *Chin. J. Physiol.* 47 89–94. 15481791

[B98] Xiong Y. Chen D. Yu C. Lv B. Peng J. Wang J. (2015). Citrus nobiletin ameliorates experimental colitis by reducing inflammation and restoring impaired intestinal barrier function. *Mol. Nutr. Food Res.* 59 829–842. 10.1002/mnfr.201400614 25655748

[B99] Xu Q. Yi L. Pan Y. Wang X. Li Y. Li J. (2008). Antidepressant-like effects of the mixture of honokiol and magnolol from the barks of *Magnolia officinalis* in stressed rodents. *Prog. Neuropsychopharmacol. Biol. Psychiatry* 32 715–725. 10.1016/j.pnpbp.2007.11.020 18093712

[B100] Yao J. Lu Y. Zhou J. P. (2008). Preparation of nobiletin in self-microemulsifying systems and its intestinal permeability in rats. *J. Pharm. Pharm. Sci.* 11 22–29. 10.18433/J3MS3M 18801304

[B101] Yu S. Yan R. Liang R. Wang W. Yang B. (2012). Bioactive polar compounds from stem bark of Magnolia officinalis. *Fitoterapia* 83 356–361. 10.1016/j.fitote.2011.11.020 22155594

[B102] Yu X. Xie J. Wang Y. Li Y. Mo Z. Zheng Y. (2015). Selective antibacterial activity of patchouli alcohol against *Helicobacter* pylori based on inhibition of urease. *Phytother. Res.* 29 67–72. 10.1002/ptr.5227 25243578

[B103] Zhang B. Wang X. Li S. (2013). An integrative platform of TCM network pharmacology and its application on an herbal formula, Qing-Luo-Yin. *Evid. Based Complement. Alternat. Med.* 2013:456747. 10.1155/2013/456747 23653662 PMC3638581

[B104] Zhang J. Li Y. Zhang L. Wang J. Yang Y. Zhang S. (2015). Systems pharmacology dissection of anti-inflammatory mechanism for medicinal herb Folium Eriobotryae. *Int. J. Mol. Sci.* 2 2913–2941. 10.3390/ijms16022913 25636035 PMC4346873

[B105] Zhang W. Li Y. Wang X. Tian F. Cao H. Wang M. (2005). Effects of magnolol and honokiol derived from traditional Chinese herbal remedies on gastrointestinal movement. *World J. Gastroenterol.* 11 4414–4418. 10.3748/wjg.v11.i28.4414 16038044 PMC4434672

[B106] Zheng X. Zhang X. Sheng X. Yuan Z. Yang W. Wang Q. (2010). Simultaneous characterization and quantitation of 11 coumarins in Radix Angelicae Dahuricae by high performance liquid chromatography with electrospray tandem mass spectrometry. *J. Pharm. Biomed. Anal.* 51 599–605. 10.1016/j.jpba.2009.09.030 19879083

[B107] Zhong Y. Yi Y. Yuan C. (2015). Analysis of the role of high performance liquid chromatography in the determination of traditional Chinese Medicine. *J. Tibet Univ.* 1 115–122.

